# *COL4A5*-p.Gly624Asp is the Predominant Variant in Europe Associated With a Mild Alport Syndrome Phenotype

**DOI:** 10.1016/j.ekir.2025.02.031

**Published:** 2025-03-06

**Authors:** Bastian M. Krüger, Annika Jens, Anna Neuhaus, Jasmina Ćomić, Riccardo Berutti, Jonathan de Fallois, Friederike Petzold, Johannes Münch, Jan Kowald, Tom H. Lindner, Klemens Budde, Klara K. Brüning, Julia Thumfart, Jacob Haas, Carolin B. Brigl, Kerstin Amann, Velibor Tasic, Nora Abazi-Emini, Valbona Nushi-Stavileci, Jovana Putnik, Nataša Stajić, Evelyn Seelow, Charlotte Hammett, Kai-Uwe Eckardt, Korbinian M. Riedhammer, Eva V. Schrezenmeier, Julia Hoefele, Jan Halbritter

**Affiliations:** 1 Division of Nephrology, Department of Internal Medicine, University Hospital Leipzig, Leipzig, Germany; 2 Department of Nephrology and Medical Intensive Care, Charité - Universitätsmedizin Berlin, Berlin, Germany; 3 Institute of Human Genetics, Klinikum rechts der Isar, Technical University of Munich, TUM School of Medicine and Health, Munich, Germany; 4 Department of Pediatric Gastroenterology, Nephrology and Metabolic Diseases, Charité - Universitätsmedizin Berlin, Berlin, Germany; 5 Department of Nephropathology, Institute of Pathology, Friedrich-Alexander-University Erlangen-Nürnberg, Erlangen, Germany; 6 University Children's Hospital, Medical Faculty of Skopje, Skopje, North Macedonia; 7 Pediatric Clinic, University Clinical Center of Kosovo, Prishtina, Kosovo; 8 Department of Nephrology, Institute for Maternal and Child Health Care of Serbia “Dr Vukan Čupić”, Faculty of Medicine, University of Belgrade, Belgrade, Serbia; 9 Department of Nephrology, Klinikum rechts der Isar, Technical University of Munich, TUM School of Medicine and Health, Munich, Germany; 10 Division of Nephrology, Department of Pediatrics, Boston Children's Hospital, Harvard Medical School, Boston, Massachusetts, USA; 11 Institute of Human Genetics, University Hospital, Ludwig-Maximilians-University, Munich, Germany

**Keywords:** alport syndrome, chronic kidney disease, collagen type IV, COL4A3, COL4A4, COL4A5

## Abstract

**Introduction:**

Pathogenic variants in *COL4A3–5* are common causes of inherited kidney disease. The clinical presentation extends from classical Alport syndrome (AS) to focal segmental glomerulosclerosis (FSGS) without extrarenal manifestation. In this study, we aimed to assess the genetic and phenotypic spectrum, along with the associated natural histories, in a cohort of patients with AS from 3 tertiary centers in Central Europe.

**Methods:**

A total of 210 patients with disease causing variants in one of the *COL4A3–5* genes were characterized and evaluated for genotype-phenotype correlations. In addition, 48 *COL4A5*-p.Gly624Asp carriers were analyzed for replication and pooled analysis.

**Results:**

*COL4A5*-p.Gly624Asp was by far the most common variant, accounting for 16% of all genetic diagnoses. These patients presented with overall milder renal phenotypes than patients with other *COL4A5* missense variants and *COL4A5* glycine-missense variants after age- and sex-matching. In patients lacking a wild-type allele (X-Linked AS [XLAS] males and autosomal recessive AS [ARAS]), histological AS was most frequently observed in kidney biopsies, and truncating variants were associated with increased disease severity. Conversely, in patients with a wild-type allele present (XLAS females and autosomal dominant AS [ADAS]), FSGS was more frequently observed. Predicted protein truncation was not inferior to missense alterations in terms of renal survival.

**Conclusion:**

The predominance of the European *COL4A5* founder variant p.Gly624Asp allowed for the creation of the largest cohort of patients with an identical Alport variant to date, confirming the more favorable renal prognosis specific to this amino acid change. Allelic and gene dosage effects drive phenotypic differences and should be incorporated into future risk models.

## Graphical abstract

**Figure:**
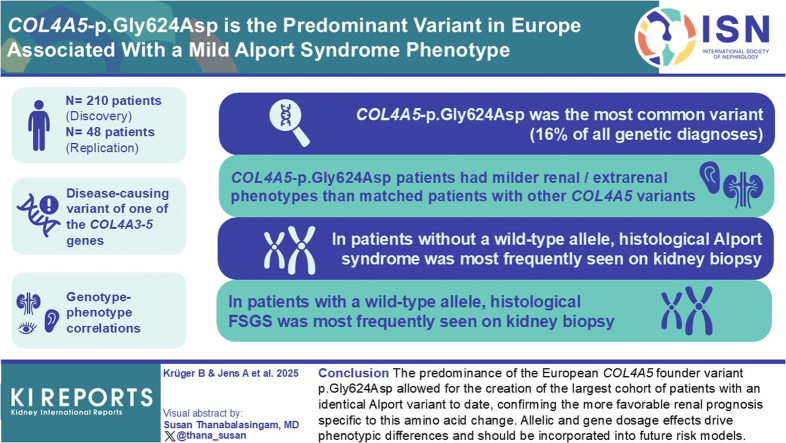

Alport syndrome (AS) because of disease-causing variants in the *COL4A3*, *COL4A4*, and *COL4A5* genes,^1^, ^2^, ^3^, ^4^ encoding the collagen IV chains at the glomerular basement membrane (GBM)^5^ has been recognized as one of the most frequent entities among hereditary nephropathies. The polypeptide chains feature collagenous domains with repetitive glycine-X-Y triplets. Glycine, positioned at every third residue, plays a pivotal role in the formation of the triple helix structure of collagen.^6^ Periodically, these sequences are interspersed with nonhelical regions or noncollagenous domains for structural flexibility^7^ and assemble into heterotrimers forming the 3-dimensional collagen IV network of the GBM.^8^^,^^9^

Although disease-causing variants in *COL4A5* lead to XLAS,^10^^,^^11^ variants in *COL4A3* and *COL4A4* result in the autosomal form, both in a biallelic (ARAS)^12^^,^^13^ and monoallelic (ADAS) fashion.^14^^,^^15^ Rarely, coexisting diagnostic variants within the *COL4A3–5* genes can be observed and are then classified as digenic AS.^16^ The terminology based on the genotype has evolved to better reflect the diversity of genetic causes and disease progression.^17^ However, frequency, penetrance, and nomenclature remain subjects of ongoing debate.

The clinical presentation of classical AS is characterized by persistent microscopic hematuria, proteinuria, and progressive loss of glomerular filtration rate (GFR), based on typical ultrastructural alterations of the GBM and accompanied by extrarenal manifestations such as bilateral sensorineural hearing impairment and ocular abnormalities.^18^, ^19^, ^20^ This profile is typically observed in males with XLAS^21^^,^^22^ as well as in both males and females with ARAS. In contrast, patients with ADAS present a broad clinical spectrum, ranging from mild courses (previously referred as to thin basement membrane nephropathy) to kidney failure (KF).^23^^,^^24^ Disease progression to late-onset KF (aged > 50 years) has been reported in up to 20% of affected individuals probably related to the development of FSGS.^25^^,^^26^ Similarly, female patients with XLAS usually display less severe symptoms but remain susceptible to progressive kidney disease.^27^^,^^28^ For XLAS, genotype-phenotype correlations indicate that truncating variants are associated with a more severe clinical phenotype, with rapid disease progression and earlier onset of KF compared with nontruncating, missense variants.^23^^,^^24^^,^^29^^,^^30^ Among the latter, a specific founder variant in *COL4A5,* namely c.1871G>A, p.Gly624Asp (in short *COL4A5*-p.Gly624Asp; https://www.ncbi.nlm.nih.gov/clinvar/RCV000021334/), has previously been reported in Eastern and Central Europe and was suggested to function as a hypomorph.^31^, ^32^, ^33^, ^34^, ^35^, ^36^ Studies found this variant to result in an average delay of KF by 20 to 30 years compared with other disease-causing *COL4A5* variants.^35^ However, particularly males with the p.Gly624Asp variant remain at significant risk for KF.

The expanding clinical spectrum of AS requires individualized counseling and tailored management. With new therapies on the horizon, risk prediction at early stages becomes very important for future clinical trial inclusion. We hypothesize that genetics can inform clinicians and their patients about the future risk of developing KF and/or extrarenal involvement.

In this retrospective multicenter study, we examined the characteristics of patients with a genetically confirmed diagnosis of type IV collagen–related nephropathy. Molecular genetic criteria were used to classify the cohort independently of the clinical phenotype. As a result, we were able to assess genotype-phenotype correlations in the largest cohort of *COL4A5*-p.Gly624Asp variant carriers to date.

## Methods

### Study Population

**Figure 1:**
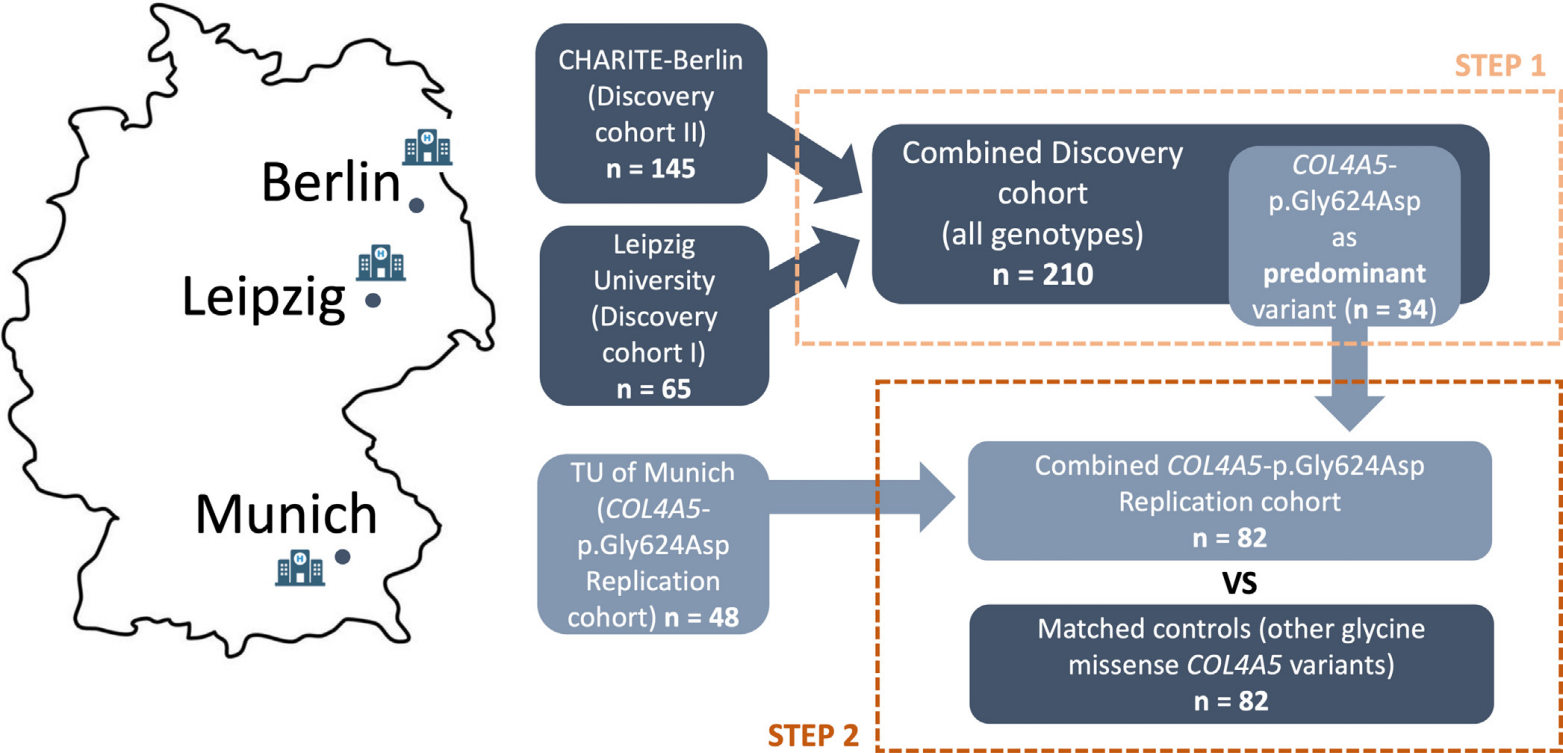
Overview of study design. In the first step, 210 patients from 2 tertiary centers (Charité Berlin and Leipzig University) with variants in one of the Alport genes were characterized clinically and genetically (discovery cohort). As a result, *COL4A5*-p.Gly624Asp (*n* = 34) turned out to be the predominant variant. In the second step, we sought for independent replication by collaboration with another tertiary center (TUM Munich), which yielded confirmatory results regarding predominance of the founder variant. Lastly, patients with the *COL4A5*-p.Gly624Asp variant were combined (*n* = 82) for joint comparison to an age-matched and sex-matched cohort with patients harboring other *COL4A5* glycine missense variants (replication cohort).

In a first step, using electronic health records and local patient databases, we screened for the diagnoses “Alport syndrome”, “thin basement membrane syndrome”, and “focal segmental glomerulosclerosis” (Figure 1). Furthermore, we examined the available genetic findings from the Departments of Nephrology at Charité Berlin and University of Leipzig, Germany, for disease-causing variants (likely pathogenic, pathogenic, and clinically suspicious [“hot”] variants of uncertain significance) in *COL4A5, COL4A4*, and *COL4A3*.^37^^,^^38^ These patients were then tested for the inclusion criteria, which were age ≥18 years and at least 1 diagnostic (i.e., disease-causing) variant in *COL4A3–5*. Patients were then contacted by phone or in writing to obtain consent to participate in the study. The study protocol was approved by the Ethics Committees of the University of Leipzig (ethics vote 059/20-ek) and Charité Berlin (EA1/104/23).

After obtaining consent, clinical data was retrospectively collected using phone interviews, a patient questionnaire, and medical records. This data was then entered into a database, which included date of birth, sex, date of inclusion, age at inclusion, ethnicity, height, weight, clinical diagnosis, renal manifestation (including creatinine at inclusion, Chronic Kidney Disease-Epidemiology Collaboration calculated estimated GFR [eGFR], proteinuria and hematuria status, need for kidney replacement therapy, age at KF), and extrarenal manifestation as well as genetic findings.

For patients for whom the last available creatinine or proteinuria measurements were conducted in the past, the age at inclusion was corrected to the corresponding age at the time of the last available measurements. An eGFR of 10 ml/min per 1.73 m^2^ was assumed for patients who had already started kidney replacement therapy. Age at KF was defined as age at initiation of kidney replacement therapy (hemodialysis, peritoneal dialysis, and kidney transplantation). Proteinuria and albuminuria status were queried for all patients but only evaluated for those whose urinary protein-to-creatinine ratio and/or urinary albumin-to-creatinine ratio data were available and for those who did not yet receive a kidney transplant. For patients who underwent a native kidney biopsy, the results were recorded and categorized into 5 categories as follows: histologic AS, FSGS, thin basement membrane nephropathy, immune-mediated glomerular disease (including minimal change disease, membranous nephropathy, and IgA nephropathy), and other (including interstitial nephritis, thrombotic microangiopathy, normal and unspecified findings). Histologic AS was diagnosed based on the ultrastructural presence of GBM lamellation, split GBM, and basket weave–like appearance. The histological diagnoses were made by a board-certified renal pathologist.

Regarding extrarenal manifestations, data was collected via questionnaire and phone interviews. Not all patients underwent audiometric testing and only for a minority was it possible to obtain data from structured ophthalmologic examinations. We therefore included the reporting of any ocular manifestation or any hearing loss as categorial variables in our analysis.

In a second step, patients with the specific hypomorphic variant *COL4A5*-p.Gly624Asp were additionally collected from the Institute of Human Genetics at the Klinikum rechts der Isar of the Technical University of Munich in Munich, Germany (Figure 1). This additional study was approved by the local Ethics Committees of the Technical University of Munich (#521/16 S). Clinical and phenotypic information were obtained from clinical reports, medical history, and phone interviews. In addition, a standardized questionnaire was used to evaluate clinical information. The combined *COL4A5*-p.Gly624Asp cohort was then compared with a matched cohort (comprised patients from Berlin, Leipzig, and Munich) with other *COL4A5* glycine missense variants.

### Molecular Genetic Findings

Description of genetic findings included mode of inheritance (XLAS, ADAS, ARAS, or digenic/complex), zygosity, type of disease-causing variant and American College of Medical Genetics and Genomics (ACMG) classification.^39^ Additionally, the variant classification of the Association for Clinical Genomic Science (ACGS) was applied (https://www.acgs.uk.com). Diagnostic variants included clinically suspicious variants of uncertain significance, likely pathogenic variants, and pathogenic variants according to ACGM and ACGS. To harmonize genetic reporting, variants which were reported according to older transcripts were translated to the MANE-Select transcript, that is, NM_033380.3 for *COL4A5*, NM_000092.5 for *COL4A4*, and NM_000091.5 for *COL4A3*.

### Statistical Analysis and Graphical Visualization

Statistical analysis was performed using Graph Pad Prism (Version 10.1.1, GraphPad Software, Boston, MA, www.graphpad.com). As part of the descriptive statistics, frequencies and percentages were calculated for categorical variables, whereas the mean ± SD were calculated for continuous variables. To compare different subgroups, Fisher exact test was used for categorical variables. Unpaired *t* tests were used for continuous variables with a normal distribution. To compare proteinuria and albuminuria, the initial values were logarithmized (log_10_) and then subjected to an unpaired *t* test. The significance level was set at 0.05 (2-sided).

## Results

**Figure 2:**
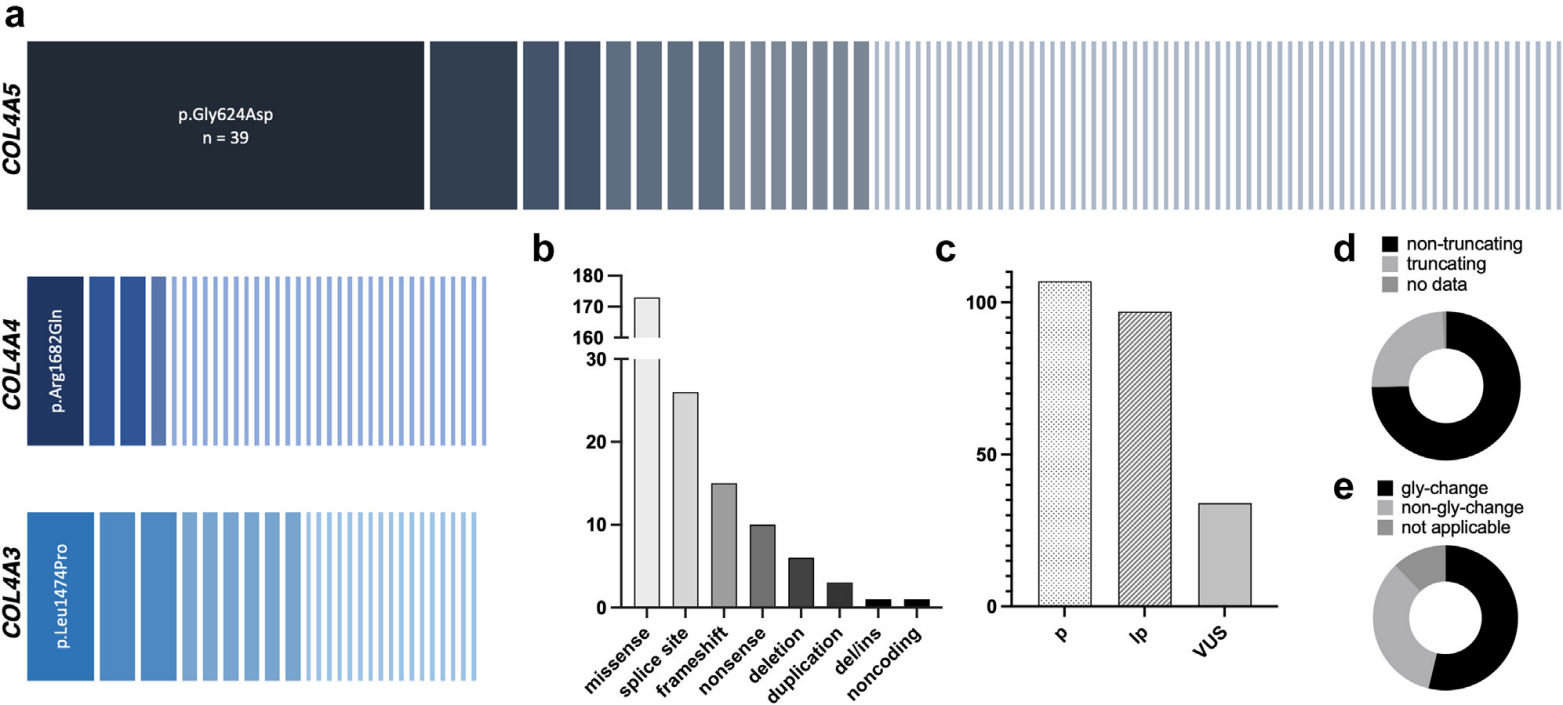
Genetic characteristics of all disease-causing variants found (*n* = 238) in the combined discovery cohort. (a) Frequency of different variants in the genes *COL4A5*, *COL4A4* and *COL4A3*, respectively. The length of a bar symbolizes the frequency of individual variants. (b) Type of disease-causing variant. (c) Frequency of pathogenic (P), likely pathogenic (LP) and clinically suspicious (“hot”) variants of uncertain significance (VUS) according to the American College of Medical Genetics and Genomics/Association for Clinical Genomic Science classifications. (d) Frequency distribution of truncating versus nontruncating variants. (e) Frequency distribution of glycine missense changes versus nonglycine missense changes.

In the first step, 210 patients (the discovery cohort) were enrolled from 2 centers (Charité Berlin/ University of Leipzig), harboring a total of 238 diagnostic variants (Figure 2). Among them, 113 were male and 97 were female. The average age was 45.4 years (SD: ± 14.5). Most patients were of European ancestry (90%), whereas 11 patients (5%) were of Middle Eastern descent. The mean body mass index was 25.9 kg/m^2^(SD: ± 5.8).

### Molecular Genetic Findings of Combined Discovery Cohort

Of the patients, 137 (65%) had a disease-causing variant in *COL4A5* and were therefore classified as having XLAS. Forty-three patients (20%) exhibited a heterozygous variant in either *COL4A4* or *COL4A3* and were defined as ADAS. In 14 patients (7%), biallelic deleterious variants in *COL4A4* or *COL4A3* led to the molecular diagnosis of ARAS. In 16 patients (8%), variants were found in > 1 of the 3 Alport genes (Supplementary Table S1).

Of the 238 disease-causing variants found in total (Figure 2a), most were missense variants (73%), followed by splice site variants (11%), frameshift (6%), and nonsense (4%) variants. In addition, there were copy number variations in terms of 9 deletions (4%) and 3 duplications (1%). In 1 patient, we detected an indel variant (c.4207delinsCC) in *COL4A4* and 1 pathogenic variant affecting a noncoding area of *COL4A5* (c.3791-41A>G) was observed in another patient (Figure 2b). Remarkably, a single *COL4A5* missense variant, the founder variant p.Gly624Asp, accounted for 16% (39 of 238) of all variants and 16% of all diagnoses (34 of 210) (Supplementary Table S7).

One hundred seven variants (45%) were classified as pathogenic, 97 variants (41%) as likely pathogenic, and 34 (14%) as clinically suspicious variants of uncertain significance (Figure 2c). Nontruncating glycine missense changes accounted for > 50% of all variants in *COL4A3-5* (Figure 2d and e).

### Clinical Characteristics of the Combined Discovery Cohort

The mean eGFR in our cohort was 40 ml/min per 1.73 m^2^ (SD: ± 41). A total of 124 patients (59%) had reached KF. Overall, the mean age at KF onset was 35.8 years (SD: ± 14.0). In XLAS, renal outcomes differed significantly between men and women, as expected. Here, 63 of the 80 XLAS males had reached KF (79%), whereas 18 of the 56 XLAS females (32%) suffered from KF. Mean age at reaching KF was 29.8 years (SD: ± 10.05) for XLAS-men and 46 years (SD: ± 16.5) for XLAS-women. Likewise, patients with ARAS suffered more often and earlier from KF compared with patients with ADAS. (Supplementary Table S1).

A total of 90 patients (43%) were kidney transplant recipients. Of those, the majority (*n* = 55, 61%) were patients lacking a wild-type allele (XLAS males and ARAS), 28% (*n* = 25) harboring a wild-type allele (XLAS females and ADAS), and 10 (11%) exhibited digenic or complex inheritance.

**Figure 3:**
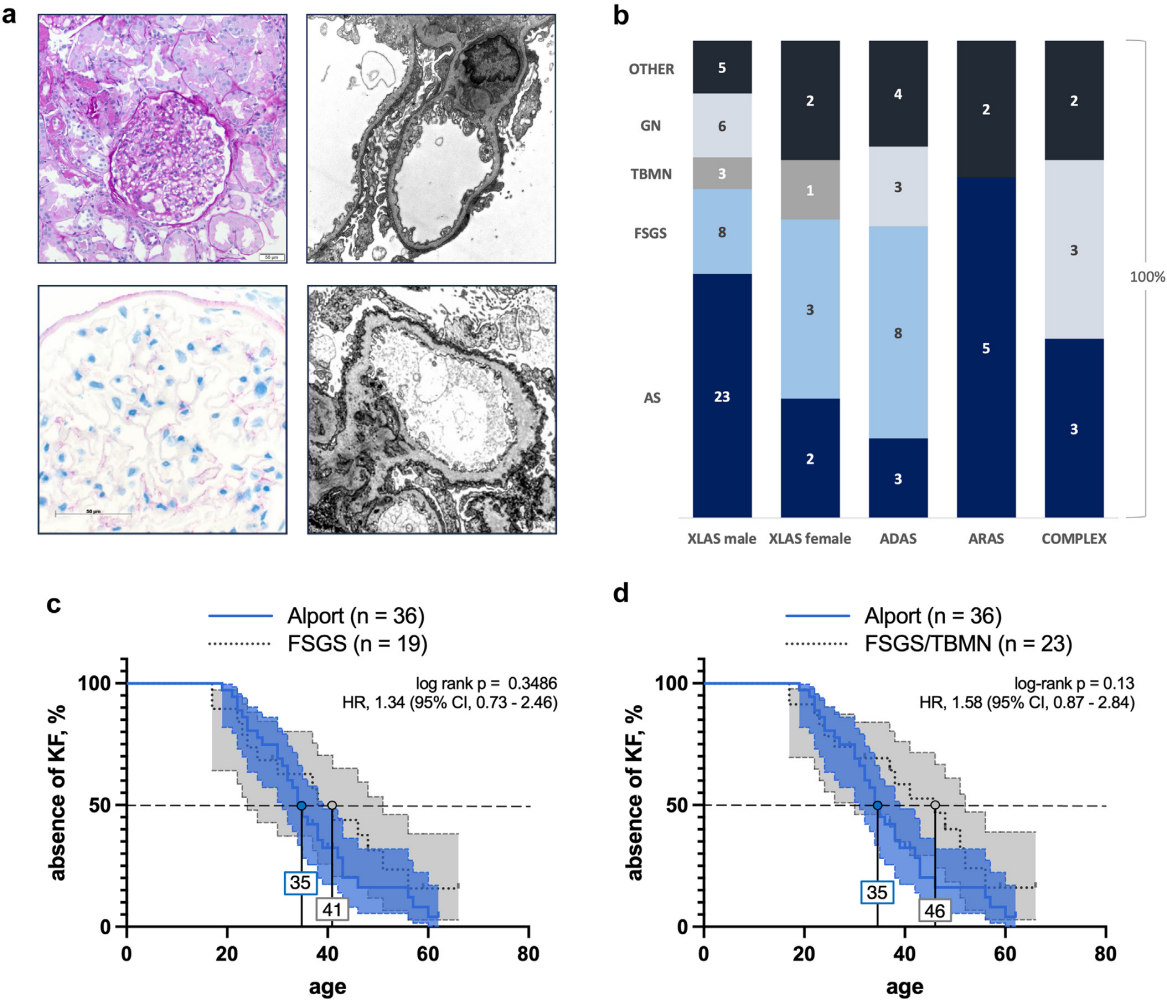
Histological findings on native kidney biopsies in the combined discovery cohort. (a)Top left: light microscopy of a glomerulus afflicted with FSGS from a patient harboring the variant c.1030-1G>C in the *COL4A4* gene; top right: electron microscopy, × 20,000, from the same patient revealing primary FSGS; bottom left: *COL4A5* immunohistochemistry of a patient harboring the compound heterozygous variants p.Gly637Arg and p.Leu1474Pro in the gene *COL4A3* shows marked deficiency of staining. Bottom right: electron microscopy, × 20,000, of the same patient shows capillary with thickening and lamellation of glomerular basement membrane. (b) Histological entities according to inheritance. Other includes interstitial nephritis, thrombotic microangiopathy, normal and unspecified findings. (c) Renal survival showed no significant discrimination when comparing patients with histological Alport syndrome to those with FSGS and (d) neither to those with FSGS or TBMN. ADAS, autosomal dominant Alport syndrome; ARAS, autosomal recessive Alport syndrome; AS, Alport syndrome; FSGS, focal segmental glomerulosclerosis; GN, immune-mediated glomerular disease (including minimal change disease, membranous nephropathy, and IgA nephropathy); TBMN, thin basement membrane nephropathy; XLAS, X-linked Alport syndrome.

Ninety-three patients (44%) underwent a native kidney biopsy. In 7 cases, histological data could not be retrieved so that a histological report was available in 86 cases. Of these, 36 (41.9%) were classified as histologic AS. The second most common finding on biopsy was FSGS, observed in 19 cases (22%). Among these, electron microscopy data was available for 10 cases, of which 7 exhibited ultrastructural anomalies in the GBM. In 12 cases (14%), the histology was compatible with different forms of immune-mediated glomerular disease. Histologic AS was the most common finding in patients without a wild-type allele (XLAS males and ARAS), whereas FSGS was the most common histological finding in patients with a wild-type allele (XLAS females and ADAS) (Figure 3b). Of note, there was no statistically significant difference in renal survival when comparing histological AS with FSGS and FSGS/thin basement membrane nephropathy (Figure 3c and d).

Data on pretransplantation proteinuria was available in 98 cases. Mean proteinuria was 1721 mg/g (SD: ±2704), whereas mean albuminuria was 1293 mg/g (SD: ±2312) (Supplementary Figure S1). Where data was available (114 cases), pretransplantation microscopic hematuria was present in nearly all patients (90%) (Supplementary Table S1).

Audiometry data was available in 179 subjects (85%) whereas data on eye examination was recorded in 136 cases (65%). Overall, 88 patients reported hearing loss (49%), whereas some kind of eye manifestation (including hypertensive retinopathy) was reported in 51 cases (38%).

### Genotype-Phenotype Correlations in Combined Discovery Cohort

To further investigate clinical outcomes, patients with monogenic AS were divided into different subgroups for mutual comparison as follows: presence of wildtype (*n* = 94) versus absence of wildtype (*n* = 100); among patients with XLAS, we tested the most frequent variant p.Gly624Asp (*n* = 34) versus other missense *COL4A5* variants (*n* = 65) in the discovery cohort.

#### Presence of Wildtype (XLAS Females and ADAS) Versus Absence of Wildtype (XLAS males and ARAS)

With 24 ml/min per 1.73 m^2^ (SD: ± 30.6), patients without a wild-type allele (XLAS males and ARAS) had a significantly lower mean eGFR than patients with a wild-type allele (XLAS females and ADAS) (57.4, SD: ± 44.5). This finding was consistent across different age groups (Supplementary Figure S2). Likewise, patients with a wild-type allele had significantly less proteinuria than those without. About 15% of patients with a wild-type allele did not have microscopic hematuria, significantly more than those without a wild-type allele (4%). In the absence of a wild-type allele, 71% (63 of 89) reported hearing loss, whereas in patients harboring a wild-type allele, hearing loss was present in only 23% (18 of 77). Similarly, whenever data were available, eye manifestation of any kind was reported more often in patients without the wildtype (49%; 33 of 68) than in patients with the wildtype (24%; 14 of 58) (Supplementary Table S2), in line with gene dosage effects.

**Figure 4:**
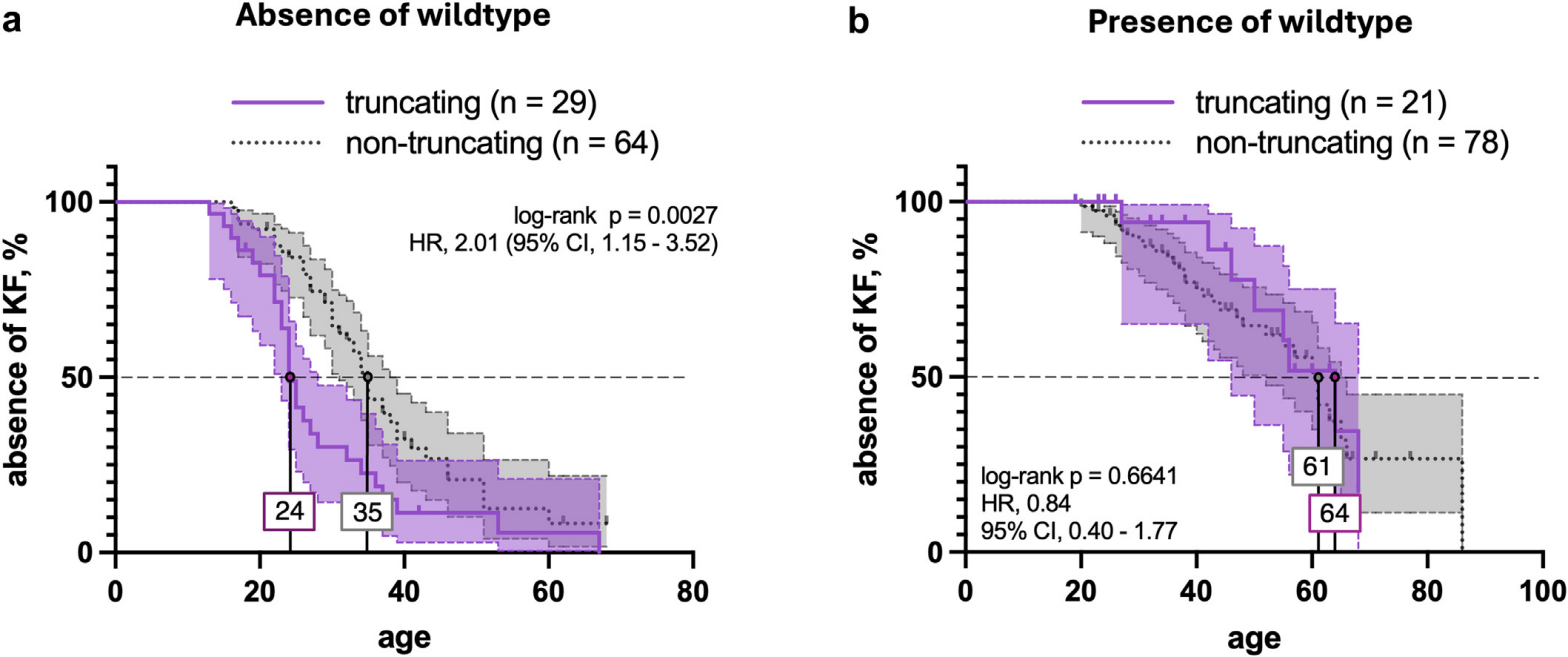
Comparative renal survival analyses of truncation versus nontruncation in the combined discovery cohort. (a) Renal survival showed significant discrimination when comparing truncating versus nontruncating variants in patients lacking the wild-type allele (XLAS males and ARAS). (b) This discrimination was not observed in patients harboring the wild-type allele, as in XLAS females and ADAS. ADAS, autosomal dominant Alport syndrome; ARAS, autosomal recessive Alport syndrome; XLAS, X-linked Alport syndrome.

Among patients without the wild-type allele, carriers of a truncating variant were significantly younger at onset of KF than those with nontruncating variants (24 vs. 35 years median renal survival) (Figure 4a). In contrast, this pattern was not observed in female XLAS and ADAS patients carrying the wildtype in *trans*. Here, median age at KF onset was 61 years for patients harboring nontruncating variants and 64 years for those with truncating variants (Figure 4b). Proteinuria and albuminuria did not differ in both groups. Analogously, *COL4A3–5* truncating variants were only linked to an increased rate of extrarenal manifestations among patients lacking the wildtype (male XLAS and ARAS) whereas this difference was not observed in patients with a preserved wild-type allele in *trans* (Supplementary Table S3 and Table S4).

#### COL4A5-Gly624Asp Versus Other COL4A5 Missense Variants

**Figure 5:**
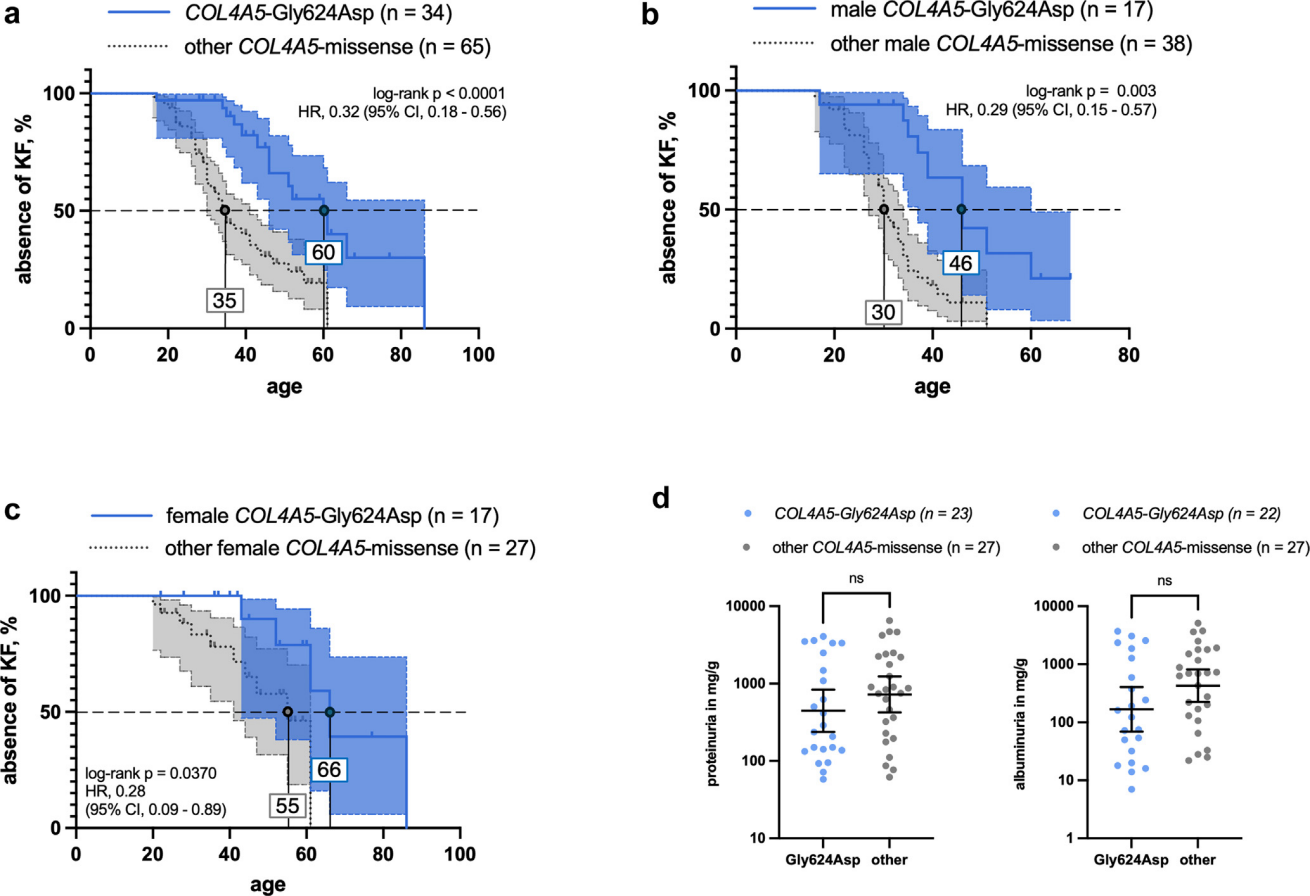
(a) Comparative renal survival analyses of carriers with *COL4A5*-p.Gly624Asp versus other *COL4A5* missense variants in the combined discovery cohort. (b and c) This difference was found in male as well as female individuals. (d) Mean proteinuria and mean albuminuria did not differ between patients carrying *COL4A5*-p.Gly624Asp and patients carrying other *COL4A5*-missense variants.

For the most common variant in the combined discovery cohort, the *COL4A5* missense variant p.Gly624Asp (*n* = 39), we sought to assess genotype-phenotype correlations under the previously reported hypomorph-hypothesis. In 34 patients, it was the only variant found (of note, 5 patients with p.Gly624Asp harbored an additional likely pathogenic or pathogenic variant in a different Alport gene). Compared with all other *COL4A5* missense variants in the cohort, patients carrying this variant had a substantially milder kidney phenotype across different age groups (Figure 5a, Supplementary Figure S3). Median age at KF was 46 years for XLAS men carrying the variant p.Gly624Asp and 30 years for those with other missense variants. For women, median age at KF was 66 years in the former group and 55 in the latter (Figure 5b and c). Mean proteinuria and albuminuria at inclusion did not differ between the 2 groups. However, extrarenal manifestations (hearing impairment and eye involvement) were less frequently reported in the group of p.Gly624Asp carriers (Supplementary Table S5).

### Comparison of an Extended Founder Cohort With Fully Matched Carriers of Other *COL4A5* Glycine Missense Variants

To increase the sample size and gain a better understanding of the variant-specific clinical picture associated with the *COL4A5* founder variant p.Gly624Asp, we included other 48 patients from a replication cohort (TUM Munich), in which p.Gly624Asp was also found to be the most frequent single disease-causing AS variant. By combining cohorts, we analyzed data from 94 patients with this founder variant. Because 12 patients showed an additional diagnostic variant in another Alport gene, we analyzed a core cohort of 82 patients. Moreover, for most accurate comparison, we established an age-matched and sex-matched cohort of patients with other glycine missense variants in *COL4A5* (Supplementary Table S6).

**Figure 6:**
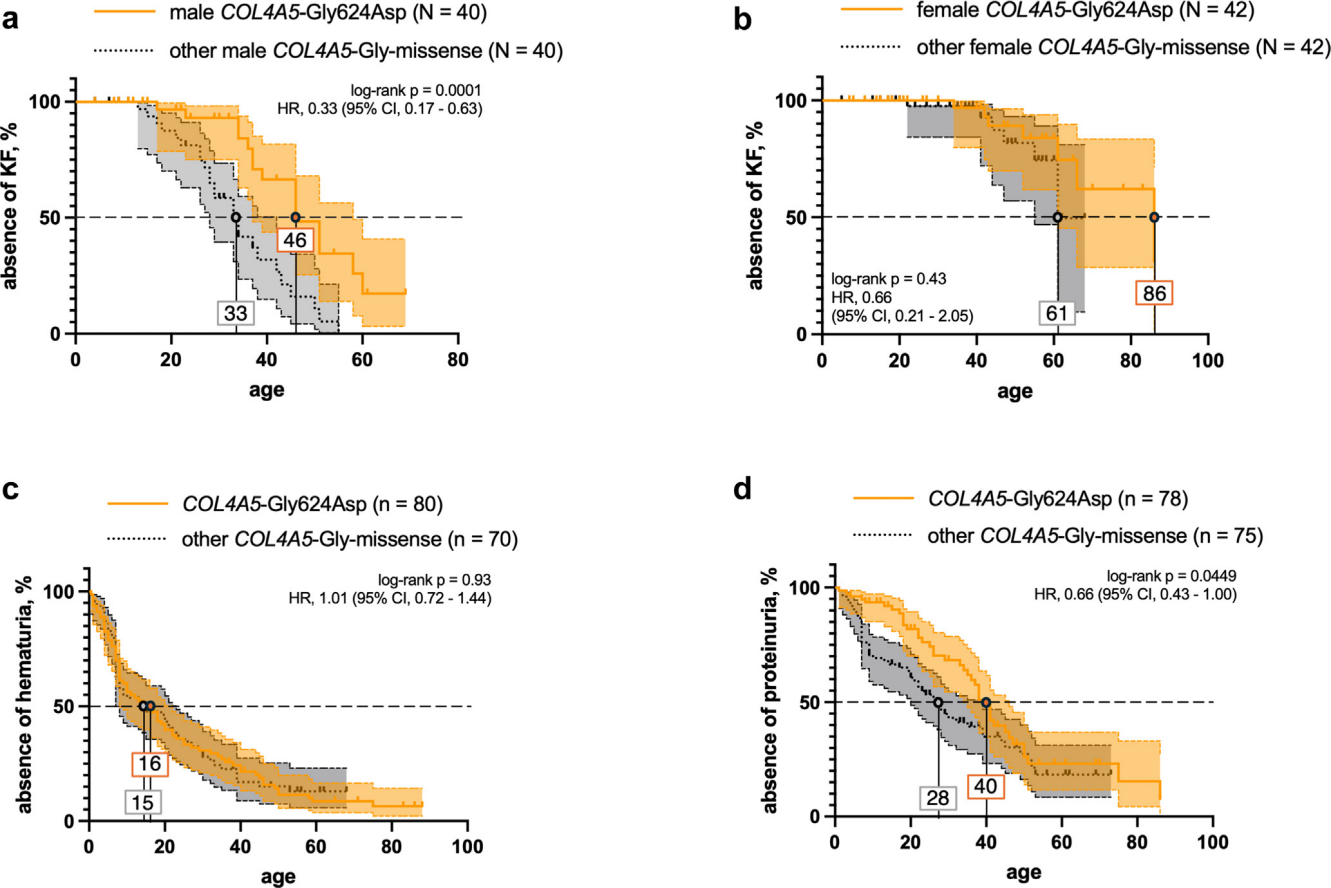
Comparative renal survival analyses of carriers with *COL4A5*-p.Gly624Asp versus matched carriers with other *COL4A5* Gly-missense variants in the combined replication cohort. (a) Renal survival showed significant discrimination when comparing male individuals with the missense variant *COL4A5*-p.Gly624Asp with individuals with other *COL4A5* glycine missense variants. (b) This difference was not found in female individuals. (c) Age at onset of hematuria was reported similar in both groups. (d) Onset of proteinuria was reported later in patients with *COL4A5*-p.Gly624Asp.

Here, we could recapitulate the more favorable renal course for males carrying *COL4A5*-p.Gly624Asp (Figure 6a). Median age at KF onset was 46 years, compared with 33 years for patients with other glycine missense variants in *COL4A5*. In females, this effect was no longer statistically significant, but appeared more favorable, matching the previous bicentric cohort-analysis (Figure 6b). Furthermore, there was no difference in age at onset of hematuria (Figure 6c). Despite insufficient data to compare mean proteinuria in this extended cohort, age at onset of proteinuria was significantly delayed upon *COL4A5*-p.Gly624Asp (Figure 6d). Furthermore, the overall frequency of kidney cysts did not differ (Supplementary Figure S4), a feature that was recently associated with AS, notably with heterozygous *COL4A3* and *COL4A4* variants.^40^ Finally, prevalences of extrarenal (eye or ear) manifestations were less frequent in carriers of p.Gly624Asp, but turned out nonsignificant because of smaller sample sizes upon Gly-, age-, and sex-matching (Supplementary Table S6).

## Discussion

The frequent detection of deleterious *COL4A3–5* variants in the general population has led to AS being recognized as one of the most common inherited kidney diseases.^41^ However, some variants contributing to the prevalence of AS are hypomorphic, with *COL4A5*-p.Gly624Asp being the most prominent example. Since its identification as an Eastern European founder variant in the Polish National Registry in 2021,^35^ little additional evidence has been gathered describing the natural history of patients carrying this specific variant.^36^ Because previous data was based on only 51 pediatric patients and renal survival analyses were conducted with data from telephone surveys in adult relatives, we sought to confirm the hypomorphic nature in a better characterized adult AS cohort. A key value of our study is the sample size and in-depth characterization of 82 mainly adult *COL4A5*-p.Gly624Asp carriers, constituting the largest Alport founder cohort to date. Our findings largely confirm the attenuated clinical course associated with p.Gly624Asp. Beyond the previous publication, however, we were able to run endpoint analyses with genetically and clinically matched control groups for more systematic comparison: First, a *COL4A5*-non-p.Gly624Asp missense cohort and second, a cohort of age-matched and sex-matched *COL4A5*-Glycine-missense carriers (other than p.Gly624Asp). Interestingly, the group differences, although less pronounced in the latter comparison remained significant throughout most analyses, indicating a variant-specific effect on *COL4A5* protein function. Although the onset of hematuria did not differ, onset of proteinuria and KF were significantly delayed in males (and trended in females) underlining the more favorable renal prognosis. In addition, we provide evidence for milder extrarenal affection in terms of hearing impairment and eye involvement, although the fully matched cohort still was not large enough to yield statistical significance (Supplementary Table S6). Consequently, these findings have direct implications for genetic counseling and would provide one piece of a holistic risk modeling in XLAS together with established risk factors such as amount of proteinuria and onset of arterial hypertension. Renal risk stratification at early stages is key for assessing the need of future trial inclusion with many new therapeutical agents being on the horizon.

Founder variants, such as in *UMOD*^42^ or *PKD2*,^43^ provide the unique opportunity to assess allelic mechanisms, inform genetic risk stratification, and study disease variability.

Beyond the founder variant, we employed the concept of “absence and presence of wildtype" for comparative analyses among patients with ADAS, ARAS, or XLAS of both sexes. By doing so, we were able to replicate the previously reported difference regarding truncating variants leading to earlier loss of kidney function than nontruncating variants in male patients with XLAS and ARAS. However, in the presence of wildtype (female patients with XLAS and patients of both sexes with ADAS), no intergroup difference was observed in terms of KF. This observation evokes the notion of a dominant-negative effect such that the wild-type allele may be perturbed by the expressed missense change rather than by a nonexpressed early truncation. Histologically, the presence of wildtype was also more frequently associated with FSGS, whereas absence of wildtype correlated with classical ultrastructural AS changes such as thinning and splitting of the GBM upon electron microscopy (Figure 3).

For the observed phenotypic and mechanistic differences, we suggest using the wildtype–related distinction of AS in clinical practice. Merging males with XLAS and all patients with ARAS into one group and females with XLAS and all patients with ADAS into another embraces the striking gene dosage effects and considers differences in terms of loss-of-function and potential dominant-negative effects that are more likely to be missed by current variant prioritization strategies.^44^ However, phenotypic differences because of X-inactivation have to be considered when grouping XLAS females with patients with ADAS. In this sense, it should also be discussed whether terms other than "Alport Syndrome" should be used in clinical practice. A term such as "Alport Spectrum Disorder"^45^ might be more appropriate to encompass the heterogeneous presentations and be less stigmatizing.

Limitations of our study comprise the retrospective character with missing values and nonstandardized data acquisition, specifically on extrarenal disease manifestations, where complete eye and ear assessments were only available in a minority of cases. Moreover, the data acquisition process did not permit us to account for potential disease-modifying factors, such as hypertension, diabetes, episodes of acute kidney injury, medications, or lifestyle-related variables. These factors could significantly influence disease progression and outcomes, potentially confounding the analyses.

The relatively homogeneous ethnicity allowed us to recruit many individuals with the identical founder variant, which for instance was less reported on the British Island (Genomics England - GEL). Interestingly, *COL4A5*-p.Gly624Asp was also found to be the most frequent Alport-variant in the American MyCode cohort (*n* = 48) from the state of Pennsylvania where self-reported German ancestry accounts for 26.1% of the total population.^46^ Similar to our findings, these patients presented with fewer incidences of KF and hearing loss compared with patients with other likely pathogenic *COL4A5* variants, indicating lower expressivity.

In summary, we provide new evidence for differential allelic effects in AS. Patients with the predominating European *COL4A5*-p.Gly624Asp variant have a better renal and likely extrarenal prognosis than their counterparts with other forms of XLAS. Future studies are needed to develop prognostic scores that incorporate this genetic information to inform clinicians and their patients about the risk of rapid progression and indication for specific treatment beyond inhibitors of the renin-angiotensin system.

## Disclosure

All the authors declared no competing interests.

## Supplementary Material

Supplementary File (PDF) **Figure S1.** Estimated glomerular filtration rate and proteinuria across age groups in the combined discovery cohort. **Figure S2:** Comparison of estimated glomerular filtration rate and proteinuria across age groups in patients with absence of a wild-type allele versus presence of a wild-type allele. **Figure S3.** Comparison of estimated glomerular filtration rate and proteinuria across age groups in patients with the *COL4A5*-p.Gly624Asp variant versus patients with other *COL4A5* missense variants. **Figure S4.** Overall occurrence of kidney cysts in the extended cohort. **Table S1**. Demographic and clinical characteristics of patients with Alport spectrum disorders in the combined discovery cohort. **Table S2.** Clinical characteristics of patients with monogenic Alport syndrome and absence of a wild-type allele versus patients with presence of a wild-type allele in the discovery cohort. **Table S3.** Truncating versus nontruncating variants in patients with monogenic Alport syndrome and presence of a wild-type allele. **Table S4.** Truncating versus nontruncating variants in patients with monogenic Alport syndrome and absence of a wild-type allele. **Table S5.** Demographic and clinical characteristics of patients with missense variants in *COL4A5* from the bicentric discovery cohort. **Table S6.** Demographic and clinical characteristics of the *COL4A5*-p.Gly624Asp replication cohort and of the corresponding control group. **Table S7.** List of all variants (*N*= 143) from the combined discovery cohort of Leipzig and Berlin.

## Footnotes

Supplementary File (PDF)
**Figure S1.** Estimated glomerular filtration rate and proteinuria across age groups in the combined discovery cohort. **Figure S2:** Comparison of estimated glomerular filtration rate and proteinuria across age groups in patients with absence of a wild-type allele versus presence of a wild-type allele. **Figure S3.** Comparison of estimated glomerular filtration rate and proteinuria across age groups in patients with the *COL4A5*-p.Gly624Asp variant versus patients with other *COL4A5* missense variants. **Figure S4.** Overall occurrence of kidney cysts in the extended cohort. **Table S1.** Demographic and clinical characteristics of patients with Alport spectrum disorders in the combined discovery cohort. **Table S2.** Clinical characteristics of patients with monogenic Alport syndrome and absence of a wild-type allele versus patients with presence of a wild-type allele in the discovery cohort. **Table S3.** Truncating versus nontruncating variants in patients with monogenic Alport syndrome and presence of a wild-type allele. **Table S4.** Truncating versus nontruncating variants in patients with monogenic Alport syndrome and absence of a wild-type allele. **Table S5:** Demographic and clinical characteristics of patients with missense variants in *COL4A5* from the bicentric discovery cohort. **Table S6.** Demographic and clinical characteristics of the *COL4A5*-p.Gly624Asp replication cohort and of the corresponding control group. **Table S7.** List of all variants (*N*= 143) from the combined discovery cohort of Leipzig and Berlin.
