# Supplementary material for: COL4A5-p.Gly624Asp is the Predominant Variant in Europe Associated With a Mild Alport Syndrome Phenotype
Source: Kidney Int Rep. 2025 Mar 6;10(5):1372–83. doi: 10.1016/j.ekir.2025.02.031 (PMC12142618; doi:10.1016/j.ekir.2025.02.031)
Supplement: Supplementary File (PDF) — Figure S1. Estimated glomerular filtration rate and proteinuria across age groups in the combined discovery cohort. Figure S2: Comparison of estimated glomerular filtration rate and proteinuria across age groups in patients with absence of a wild-type allele versus presence of a wild-type allele. Figure S3. Comparison of estimated glomerular filtration rate and proteinuria across age groups in patients with the COL4A5-p.Gly624Asp variant versus patients with other COL4A5 missense variants. Figure S4. Overall occurrence of kidney cysts in the extended cohort. Table S1. Demographic and clinical characteristics of patients with Alport spectrum disorders in the combined discovery cohort. Table S2. Clinical characteristics of patients with monogenic Alport syndrome and absence of a wild-type allele versus patients with presence of a wild-type allele in the discovery cohort. Table S3. Truncating versus nontruncating variants in patients with monogenic Alport syndrome and presence of a wild-type allele. Table S4. Truncating versus nontruncating variants in patients with monogenic Alport syndrome and absence of a wild-type allele. Table S5. Demographic and clinical characteristics of patients with missense variants in COL4A5 from the bicentric discovery cohort. Table S6. Demographic and clinical characteristics of the COL4A5-p.Gly624Asp replication cohort and of the corresponding control group. Table S7. List of all variants (N= 143) from the combined discovery cohort of Leipzig and Berlin. [file mmc1.pdf]

SUPPLEMENTARY MATERIAL

Table S1: Demographic and clinical characteristics of patients with Alport spectrum disorders in the combined discovery cohort.

Table S2: Clinical characteristics of patients with monogenic Alport syndrome and absence of a wildtype allele vs. patients with presence of a wildtype allele in the discovery cohort.

Table S3: Truncating vs. non-truncating variants in patients with monogenic Alport syndrome and presence of a wildtype allele.

Table S4: Truncating vs. non-truncating variants in patients with monogenic Alport syndrome and absence of a wildtype allele.

Table S5: Demographic and clinical characteristics of patients with missense variants in *COL4A5* from the bicentric discovery cohort.

Table S6: Demographic and clinical characteristics of the *COL4A5*-p.Gly624Asp replication cohort and of the corresponding control group.

Table S7: list of all variants (n = 143) from the combined discovery cohort of Leipzig and Berlin.

Figure S1: eGFR and proteinuria across age groups in the combined discovery cohort.

Figure S2: Comparison of eGFR and proteinuria across age groups in patients with absence of a wildtype allele versus presence of a wildtype allele.

Figure S3: Comparison of eGFR and proteinuria across age groups in patients with the *COL4A5*-p.Gly624Asp variant versus patients with other *COL4A5* missense variants .

Figure S4: overall occurrence of kidney cysts in the extended cohort.

	TOTAL	XLAS			ADAS	ARAS	digenic/complex*
		TOTAL	MALE	FEMALE			
n	210	137	80	57	43	14	16
age, y, mean (SD)	45,39 (±14,49)	43,70 (±14,19)	43,30 (12,46)	44,24 (16,42	50,60 (±15,65)	42,29 (±14,29)	48,50 (±10,63)
female, %	46,19	41,61	0	57	65,12	50	31,25
white, %	90,48	90,51	0	100	88,37	92,86	93,75
BMI, mean (SD)	25,86 (±5,8)	25,17 (±5,57)	24,74 (±5,53)	25,95(±5,68)	27,67 (±6,79)	25,10 (±4,53)	26,86 (±5,19)
eGFR (creatinine, CKD-EPI), mean (SD)	39,82 (41,17)	40,6 (42,29)	23,39 (30,54)	64,75 (44,83)	47,74 (42,62)	27,5 (31,92)	22,69 (28,14)
KF, n (%)	124 (59,05)	81 (59,12)	63 (78,75)	18 (31,58)	21 (58,84)	9 (64,29)	13 (81,25)
KF age, mean (SD)	35,77 (±14.0)	33,38 (±13,5)	29,78 (±10,05)	46,00 (±16,50)	43,8 (±15,1)	34,3 (±13,6)	38,6 (±11,3)
kidney transplant (%)	90 (42,9)	60 (43,8)	47 (58,75)	13 (22,81)	12 (27,9)	8 (57,1)	10 (62,5)
kidney biopsy (%)	93 (44,29)	58 (42,34)	47 (58,75)	11 (19,3)	19 (44,19)	8 (57,14)	8 (50,0)
proteinuria, data available, n	98	66	26	40	20	6	6
proteinuria, mean<br>SD	1721,17<br>2704,70	1500,88<br>2031,85	2816,42<br>2572,43	645,78<br>857,36	1280,95<br>1677,20	3312,67<br>2358,28	4020,33<br>7823,01
albuminuria, data available, n	93	63	24	39	19	6	5
albuminuria, mean<br>SD	1293,96<br>2312,14	1073,35<br>1552,35	2081,63<br>1976,94	452,87<br>727,32	900,63<br>1390,56	2621,67<br>1983,96	3975,00<br>7671,61
hematuria, data available, n	114	74	30	44	28	6	6
hematuria, n (%)	103 (90,35)	68 (91,89)	29 (96,67)	39 (88,64)	24 (85,71)	6 (100)	5 (83,33)
hearing impairment, data available, n	179	118	77	41	33	12	16
reported hearing impairment, n (%)	88 (49,16)	63 (53,39)	54 (70,13)	9 (21,95)	9 (27,27)	9 (75)	7 (43,75)
eye manifestation, data available, n	136	94	60	34	24	7	11
reported eye manifestation, n (%)	51 (37,5)	33 (35,11)	27 (45)	6 (17,65)	8 (33,33)	6 (75)	5 (45,45)

Table S1: Demographic and clinical characteristics of patients with Alport spectrum disorders in the combined discovery cohort. BMI, body-mass-index; XLAS, X-linked Alport syndrome; ADAS, autosomal dominant Alport syndrome; ARAS, autosomal recessive Alport syndrome. KF, kidney failure. For patients who already reached KF the eGFR was set to 10 ml/min. *patients with a pathogenic (p) or likely pathogenic (lp) variant in one of the *COL4A3-5* genes and at least one other p, lp or clinically suspicious ("hot") variant of uncertain significance in another *COL4A3-5* gene were classified having digenic/complex inheritance.

Table S2: Clinical characteristics of patients with monogenic Alport syndrome and absence of a wildtype allele vs. patients with presence of a wildtype allele. BMI, body-mass-index; KF, kidney failure. For patients who already reached KF the eGFR was set to 10 ml/min.

	**absence of wildtype (XLAS male / ARAS)**	**presence of wildtype (XLAS female / ADAS)**	*p*-Value
n	94	100	-
age, y, mean (SD)	43.15 (±12,67)	46.99 (±16,32)	0.07
female, %	7,45	85	<0.0001
white, %	85 (90,42)	90 (90)	>0.99
BMI, mean (SD)	24.8 (5,35)	26,8 (6,25)	0.03
eGFR, mean (SD)	24,00 (30,61)	57,44 (44,49)	<0.0001
KF, n (%)	72 (76,6)	39 (39)	<0.0001
KF age, mean (SD)	30,35 (10,55)	44,82 (15,59)	<0.0001
kidney transplant, n (%)	55 (58,51)	25 (25)	<0.0001
kidney biopsy, n/N (%)	55/77 (71,43)	30/91 (32,97)	<0.0001
proteinuria, data, n	32	60	-
proteinuria, mean<br>SD	2909<br>2504	857,5<br>1218	<0,0001
hematuria, n/N (%)	69/72 (95,83)	78/91 (85,71)	0,0355
reported hearing impairment, n/N (%)	63/89 (70,79)	18/77 (23,38)	<0,0001
reported eye manifestation, n/N (%)	33/68 (48,53)	14/58 (24,14)	0,0057

Table S3: Truncating vs. non-truncating variants in patients with monogenic Alport syndrome and presence of a wildtype allele (XLAS female and ADAS). BMI, body-mass-index; KF, kidney failure. For patients who already reached KF the eGFR was set to 10 ml/min.

	**presence of wildtype (XLAS female & ADAS)**		
	**truncating**	**non-truncating**	*p*-Value
n	21	78	-
age, y, mean (SD)	48,10 (18,03)	46,73 (16,05)	0,7369
female, %	20 (95,24)	64 (82,05)	0,1816
white, %	18 (85,71)	71 (91,03)	0,4381
BMI, mean (SD)	27,30 (6,28)	26,54 (6,23)	0,6821
eGFR, mean (SD)	68,43 (49,43)	54,41 (43,21)	0,2376
KF, n (%)	8 (38.1)	31 (39,74)	>0.9999
KF age, mean (SD)	51 (12,99)	43,23 (16,0)	0,2131
kidney transplant, n (%)	5 (23,81)	20 (25,64)	>0.9999
kidney biopsy, n/N (%)	6/19 (31,58)	24/71 (33,8)	>0.9999
proteinuria, data, n	18	63	-
proteinuria, mean<br>SD	457,4<br>511,9	731,8<br>1202	0,9798
hematuria, n/N (%)	17/18 (94,44)	60/72 (83,33)	0,4524
reported hearing impairment, n/N (%)	6/17 (35,29)	12/59 (20,34)	0,2119
reported eye manifestation, n/N (%)	5/12 (41,67)	9/45 (20,0)	0,1440

Table S4: Truncating vs. non-truncating variants in patients with monogenic Alport syndrome and absence of a wildtype allele (XLAS male and ARAS). BMI, body-mass-index; KF, kidney failure. For patients who already reached KF the eGFR was set to 10 ml/min.

	**absence of wildtype (XLAS male & ARAS)**		
	**truncating**	**non-truncating**	*p*-Value
n	29	64	-
age, y, mean (SD)	43,97 (12,59)	42,59 (12,80)	0,6315
female, %	4 (13,79)	3 (4,69)	0,1987
white, %	25 (86,21)	59 (92,19)	0,4530
BMI, mean (SD)	24,26 (3,78)	25,06 (5,97)	0,5265
eGFR, mean (SD)	19,52 (28,80)	26,25 (31,58)	0,0719
KF, n (%)	26 (89,66)	45 (70,31)	0,0636
KF age, mean (SD)	27,50 (11,78)	31,89 (9,65)	0,0934
kidney transplant, n (%)	23 (79,31)	32 (50)	0,0116
kidney biopsy, n/N (%)	14/21 (66,67)	40/55 (72,73)	0,5867
proteinuria, data, N	22	56	-
proteinuria, mean<br>SD	1410<br>2434	1706<br>2329	0,4125
hematuria, n/N (%)	16/17 (94,12)	53/55 (96,36)	0,5601
reported hearing impairment, n/N (%)	25/29 (86,21)	38/59 (64.41)	0,0441
reported eye manifestation, n/N (%)	16/19 (84,21)	17/49 (34,69)	0,0003

Table S5: demographic and clinical characteristics of patients with missense variants in *COL4A5* from the bicentric discovery cohort. BMI, body-mass-index; KF, kidney failure. For patients who already reached KF the eGFR was set to 10 ml/min.

**XLAS – missense variants**	**p.Gly624Asp**	**Other missense**	*p*-value
n	34	65	-
age, y, mean (SD)	48.50 (15,96)	43.22 (13,02)	0.08
female, %	50	41,5	0.52
white, %	100	96.88	0.54
BMI, mean (SD)	27.31 (8.07)	24.19 (4.08)	0.08
eGFR, mean (SD)	50,50 (41,52)	35,12 (39,75)	0.075
KF, n (%)	14 (41,18)	42 (64,62)	0.03
KF, age, mean (SD)	48,07 (16,79)	31,86 (9,90)	0.0002
kidney transplant (%)	8 (25,53)	32 (49,23)	0.02
proteinuria, data, N	23	27	-
proteinuria, mean (SEM)	1139 (297,6)	1487 (328,6)	0.23
albuminuria, data, N	22	27	-
albuminuria, Mean (SEM)	766,5 (246,3)	1081 (253,8)	0.077
hematuria, n/N (%)	22/25 (88)	30/32 (93,75)	0.6446
reported hearing impairment, n/N (%)	8/18 (30.77)	34/58 (58.62)	0.03
reported eye manifestation, n/N (%)	2/24 (8,33)	17/48 (35,42)	0.02

Table S6: demographic and clinical characteristics of the *COL4A5*-p.Gly624Asp replication cohort and of the corresponding control group (patients with other COL4A5 glycine missense variants). KF, kidney failure. For patients who already reached KF the eGFR was set to 10 ml/min.

XLAS – missense variants	p.Gly624Asp	Other Gly-missense	*p*-value
n	82	82	-
age, y, mean (SD)	39,40 (21,69)	37,56 (18,47)	0.56
female, %	57,32	57,32	>0.99
eGFR, mean (SD)	75,49 (49,62)	59,69 (47,19)	0.11
KF, n (%)	18 (21,95)	28 (34,15)	0.12
KF, age, mean (SD)	46,44 (15,76)	33,96 (13,10)	0.006
kidney transplant (%)	11 (13,41)	18 (21,95)	0.22
reported hearing impairment, n (%)	16 (19,51)	25 (30,49)	0.15
reported eye manifestation, n (%)	7 (8,54)	11 (13,41)	0.45

Table S7: List of all variants (n = 143) from the combined discovery cohort of Leipzig and Berlin. The variant classification is derived from genetic reports. ACMG criteria are shown only for variants not listed in ClinVar/LOVD. For cases where the genetic evaluation was conducted prior to the publication of the ACMG criteria, a reclassification was performed (*).

Gene	reference sequence	sequence variant	predicted consequence	LOVD	gnomAD exomes allele frequency total	gnomAD exomes allele frequency european non-finnish	ClinVar clinical significance	Classification according to genetic report (ACMG)	count
COL4A3	NM_000091.5	c.1087G>A	p.Gly363Arg	not reported	0.000001201	0.000001313	likely pathogenic	lp	1
COL4A3	NM_000091.5	c.1183G>A	p.Gly395Arg	not reported	0.000001591	0.00005652	lp (1) / VUS (1)	lp	1
COL4A3	NM_000091.5	c.1408+1G>C	p.?	not reported	0.000002055	0.000000849	pathogenic	p	1
COL4A3	NM_000091.5	c.1483C>T	p.His495Tyr	5 reported	0.0009059	0.001086	VUS (5) / lb (1)	VUS	2
COL4A3	NM_000091.5	c.1637C>T	p.Pro546Leu	not reported	0.00002599	0	VUS (2) / lb (1)	VUS	2
COL4A3	NM_000091.5	c.1855G>A	p.Gly619Arg	5 reported	0.000006157	0.000006295	pathogenic/likely pathogenic	lp	2
COL4A3	NM_000091.5	c.1892G>T	p.Gly631Val	2 reported	0	0	pathogenic (2) / VUS (1)	lp	1
COL4A3	NM_000091.5	c.1908_1910delinsAGA	p.Gly637Glu	not reported	0	0	not listed	lp (PM1, PM2, PP3, PP4)	1
COL4A3	NM_000091.5	c.1909G>A	p.Gly637Arg	1 reported	0.000002737	0.000003390	likely pathogeic (1) / VUS (1)	lp	4
COL4A3	NM_000091.5	c.2083G>A	p.Gly695Arg	12 reported	0.0001712	0.0002162	p (5) / lp (3) / VUS (1)	p	4
COL4A3	NM_000091.5	c.2126-1G>C	p.?	3 reported	0	0	likely pathogenic	p	2
COL4A3	NM_000091.5	c.221C>T	p.Pro74Leu	not reported	0.00001509	0.000002546	VUS (4) / lb (1)	VUS	1
COL4A3	NM_000091.5	c.2284G>C	p.Gly762Arg	1 reported	0.000008	0	likely pathogenic	lp	1
COL4A3	NM_000091.5	c.235-1G>A	p.?	not reported	0.000001591	0.000002857	likely pathogenic	lp	1
COL4A3	NM_000091.5	c.2434G>A	p.Gly812Ser	1 reported	0,00003883	0,00004325	VUS	lp	1
COL4A3	NM_000091.5	c.3013_3038del	p.Pro1005Thrfs*13	not reported	not listed	not listed	pathogenic	p	1

Gene	reference sequence	sequence variant	predicted consequence	LOVD	gnomAD exomes allele frequency total	gnomAD exomes allele frequency european non-finnish	ClinVar clinical significance	Classification according to genetic report (ACMG)	count
COL4A3	NM_000091.5	c.304T>C	p.Ser102Pro	not reported	0,000007527	0,00001017	not listed	VUS (PM2, PP4, BP4)	1
COL4A3	NM_000091.5	c.3580del	p.Arg1194Glyfs*27	1 reported	not listed	not listed	pathogenic	p	1
COL4A3	NM_000091.5	c.361G>A	p.Gly121Ser	not reported	0,000009579	0,000004238	likely pathogenic (3), VUS (2)	p	1
COL4A3	NM_000091.5	c.4007T>C	p.Ile1336Thr	not reported	0,0000171	0,00001949	not listed	VUS (PM1, PM2, PP4, BP4)	1
COL4A3	NM_000091.5	c.4421T>C	p.Leu1474Pro	17 reported	0,0046	0,005682	likely pathogenic (4), VUS (12), benign(1), likely benign (2)	VUS	7
COL4A3	NM_000091.5	c.443G>T	p.Gly148Val	7 reported	0,00002052	0,00002373	pathogenic (2), likely pathogenic (3), VUS (1)	lp	2
COL4A3	NM_000091.5	c.4484A>G	p.Gln1495Arg	8 reported	0,009518	0,01136	benign/likely benign	VUS	1
COL4A3	NM_000091.5	c.4803del	p.Gly1602Alafs*13	2 reported	0,00004515	0,00005847	pathogenic/Likely pathogenic	p	1
COL4A3	NM_000091.5	c.4981C>T	p.Arg1661Cys	11 reported	0,0006116	0,0007289	pathogenic (7), likely pathogenic (12), VUS (2)	lp	2
COL4A3	NM_000091.5	Ex3del	p.?	not reported	not listed	not listed	not listed	lp (PP4, PVS1, PM2)	1
COL4A4	NM_000092.5	c.1030-1G>C	p.?	not reported	not listed	not listed	likely pathogenic	lp	1
COL4A4	NM_000092.5	c.1396G>A	p.Gly466Arg	3 reported	0,000008211	0,000008477	pathogenic/likely pathogenic	p	3
COL4A4	NM_000092.5	c.1451G>T	p.Gly484Val	not reported	not listed	not listed	not listed	lp	3

Gene	reference sequence	sequence variant	predicted consequence	LOVD	gnomAD exomes allele frequency total	gnomAD exomes allele frequency european non-finnish	ClinVar clinical significance	Classification according to genetic report (ACMG)	count
								(PM1, PM2, PP3, PP4)	
COL4A4	NM_000092.5	c.1505dupC	p.Gly503Trpfs*12	1 reported	0,000001591	0,000002856	not listed	p	1
COL4A4	NM_000092.5	c.1668G>T	p.Lys556Asn	not reported	0,000013	0,00001525	VUS	VUS	1
COL4A4	NM_000092.5	c.1744C>T	p.Gln582*	not reported	not listed	not listed	not listed	lp (PVS1, PM2, PP4)	1
COL4A4	NM_000092.5	c.1792C>A	p.Pro598Thr	not reported	0,000004771	0,000009566	VUS	VUS	1
COL4A4	NM_000092.5	c.1986A>T	p.Lys662Asn	3 reported	0,000002055	0,000002698	VUS	VUS	1
COL4A4	NM_000092.5	c.2102del	p.Gly701Valfs*52	not reported	not listed	not listed	not listed	lp (PVS1, PM2, PP4)	1
COL4A4	NM_000092.5	c.2165-2A>T	p.?	not reported	not listed	not listed	not listed	lp (PVS1, PM2, PP4)	1
COL4A4	NM_000092.5	c.2171G>A	p.Arg724His	not reported	0,00007389	0,00008899	VUS (1), likely benign (1)	VUS	1
COL4A4	NM_000092.5	c.2435_2436del	p.Glu812Glyfs*17	not reported	0,0000006841	0,0000008993	pathogenic	lp	1
COL4A4	NM_000092.5	c.3044G>A	p.Gly1015Glu	3 reported	0,00005883	0,00007373	pathogenic (1), likely pathogenic (2), VUS (2)	p	1
COL4A4	NM_000092.5	c.3089G>A	p.Gly1030Asp	not reported	0,00000159	0	VUS	lp	2
COL4A4	NM_000092.5	c.3688G>C	p.Gly1230Arg	not reported	not listed	not listed	not listed	lp* (PM1, PM2, PP3, PP4)	1
COL4A4	NM_000092.5	c.4082-1G>C	p.?	1 reported	0,000003183	0,000005715	not listed	lp	1
COL4A4	NM_000092.5	c.4129C>T	p.Arg1377*	7 reported	0,000008209	0,000008093	pathogenic	p	1
COL4A4	NM_000092.5	c.4207delinsCC	p.Ser1403Profs*30	not reported	not listed	not listed	not listed	p	1

Gene	reference sequence	sequence variant	predicted consequence	LOVD	gnomAD exomes allele frequency total	gnomAD exomes allele frequency european non-finnish	ClinVar clinical significance	Classification according to genetic report (ACMG)	count
								(PVS1, PM2, PP4)	
COL4A4	NM_000092.5	c.4333+1G>A	p.?	not reported	not listed	not listed	not listed	lp (PVS1, PM2, PP4)	1
COL4A4	NM_000092.5	c.4390_4401del	p.Gly1464_Leu1467del	not reported	not listed	not listed	not listed	VUS (PM2, PP4)	1
COL4A4	NM_000092.5	c.4417C>A	p.Gln1473Lys	2 reported	0,000008209	0,00001356	VUS	VUS	1
COL4A4	NM_000092.5	c.4470del	p.Tyr1491Ilefs*61	not reported	0,000004788	0,000001799	pathogenic	p	1
COL4A4	NM_000092.5	c.4523-1G>A	p.?	2 reported	not listed	not listed	pathogenic	lp	1
COL4A4	NM_000092.5	c.475C>T	p.Pro159Ser	not reported	0,00001369	0,00001441	VUS (1), likely benign (1)	VUS	1
COL4A4	NM_000092.5	c.4982T>A	p.Phe1661Tyr	1 reported	0,0002565	0,0003135	VUS (5), likely benign (1)	VUS	1
COL4A4	NM_000092.5	c.5044C>T	p.Arg1682Trp	1 reported	0,000013	0,00001356	likely pathogenic (1), VUS (2)	lp	1
COL4A4	NM_000092.5	c.5045G>A	p.Arg1682Gln	8 reported	0,00007046	0,00004407	pathogenic (1), VUS (4)	lp	6
COL4A4	NM_000092.5	c.594+1G>A	splice	1 reported	not listed	not listed	pathogenic/likely pathogenic	p	1
COL4A4	NM_000092.5	c.754G>A	p.Gly252Ser	1 reported	0,000001369	0,000002543	likely pathogenic (1), VUS (1)	lp	1
COL4A4	NM_000092.5	c.755G>A	p.Gly252Asp	not reported	0,0000006843	0,0000008994	likely pathogenic	lp	1
COL4A4	NM_000092.5	c.81_86del	p.Ile29_Leu30del	11 reported	0,00005352	0,00006461	likely pathogenic (5), VUS (2)	VUS	1
COL4A4	NM_000092.5	c.836G>T	p.Gly279Val	not reported	not listed	not listed	not listed	VUS (PM2, PP3, PP4)	1
COL4A4	NM_000092.5	c.93_94del	p.Ser32Cysfs*28	1 reported	0,0000006858	0,0000009019	pathogenic	p	1

Gene	reference sequence	sequence variant	predicted consequence	LOVD	gnomAD exomes allele frequency total	gnomAD exomes allele frequency european non-finnish	ClinVar clinical significance	Classification according to genetic report (ACMG)	count
COL4A4	NM_000092.5	Ex18-23dup	p.?	not reported	not listed	not listed	not listed	lp (PP4, PVS1, PM2)	1
COL4A4	NM_000092.5	Ex4del	p.?	not reported	not listed	not listed	not listed	lp (PP4, PVS1, PM2)	1
COL4A5	NM_033380.3	c.(?-2918_3106+?)del	Deletion Exon 34&35	not reported	not listed	not listed	not listed	lp* (PP4, PVS1, PM2)	1
COL4A5	NM_033380.3	c.1121G>A	p.Gly374Glu	2 reported	not listed	not listed	not listed	lp	1
COL4A5	NM_033380.3	c.1130G>T	p.Gly377Val	2 reported	not listed	not listed	not listed	lp	1
COL4A5	NM_033380.3	c.1217G>A	p.Gly406Asp	not reported	not listed	not listed	pathogenic	p	2
COL4A5	NM_033380.3	c.1218_1259del	p.Gln407_Gly420del	not reported	not listed	not listed	not listed	lp (PM1, PM2, PP4)	1
COL4A5	NM_033380.3	c.1226G>T	p.Gly409Val	2 reported	not listed	not listed	pathogenic	p	1
COL4A5	NM_033380.3	c.1312G>A	p.Gly438Ser	not reported	not listed	not listed	not listed	lp (PM1, PM2, PP3, PP4, PP5)	1
COL4A5	NM_033380.3	c.1390G>A	p.Asp464Asn	1 reported	not listed	not listed	not listed	VUS	1
COL4A5	NM_033380.3	c.1423+1G>A	p.?	2 reported	0,0000009399	0	pathogenic	p	4
COL4A5	NM_033380.3	c.1587+5G>C	p.?	4 reported	not listed	not listed	not listed	lp	1
COL4A5	NM_033380.3	c.1708G>A	p.Gly570Arg	not reported	not listed	not listed	likely pathogenic	lp	1
COL4A5	NM_033380.3	c.1718G>T	p.Gly573Val	2 reported	not listed	not listed	pathogenic	p	1
COL4A5	NM_033380.3	c.1773del	p.Glu592Serfs*26	not reported	not listed	not listed	not listed	p (PVS1, PM2, PP4)	1

Gene	reference sequence	sequence variant	predicted consequence	LOVD	gnomAD exomes allele frequency total	gnomAD exomes allele frequency european non-finnish	ClinVar clinical significance	Classification according to genetic report (ACMG)	count
COL4A5	NM_033380.3	c.1807G>A	p.Gly603Ser	not reported	not listed	not listed	pathogenic	p	1
COL4A5	NM_033380.3	c.1871G>A	p.Gly624Asp	26 reported	0.00004465	0.00006146	pathogenic/ likely pathogenic	p	39
COL4A5	NM_033380.3	c.2_5del	p.Met1?	not reported	not listed	not listed	not listed	lp (PVS1, PM2, PP4)	1
COL4A5	NM_033380.3	c.2023G>A	p.Gly675Ser	3 reported	not listed	not listed	pathogenic	lp	1
COL4A5	NM_033380.3	c.2041+1G>A	p.?	2 reported	not listed	not listed	not listed	p	2
COL4A5	NM_033380.3	c.2105G>A	p.Gly702Asp	not reported	not listed	not listed	not listed	lp (PM1, PM2, PP3, PP4)	1
COL4A5	NM_033380.3	c.2146+1G>A	p.?	not reported	not listed	not listed	not listed	lp (PVS1, PM2, PP4)	1
COL4A5	NM_033380.3	c.2165G>A	p.Gly722Glu	1 reported	0,000000932	0,000001211	pathogenic	p	1
COL4A5	NM_033380.3	c.2245-1G>A	p.?	3 reported	not listed	not listed	pathogenic	p	1
COL4A5	NM_033380.3	c.2246G>A	p.Gly749Asp	1 reported	not listed	not listed	likely pathogenic	lp	3
COL4A5	NM_033380.3	c.2287G>A	p.Gly763Arg	2 reported	not listed	not listed	not listed	lp	4
COL4A5	NM_033380.3	c.2348C>T	p.Pro783Leu	2 reported	0,00007655	0,00008721	VUS (1) / likely benign(2)	VUS	1
COL4A5	NM_033380.3	c.2475_2483del	p.Pro826_Gly828del	3 reported	not listed	not listed	pathogenic/ likely pathogenic	lp	1
COL4A5	NM_033380.3	c.2605G>A	p.Gly869Arg	23 reported	0,000001825	0,000002381	pathogenic/ likely pathogenic	p	1
COL4A5	NM_033380.3	c.2723G>C	p.Gly908Ala	not reported	not listed	not listed	not listed	lp (PM1, PM2, PP3, PP4)	1
COL4A5	NM_033380.3	c.2746A>G	p.Ser916Gly	6 reported	not listed	not listed	not listed	VUS	1

Gene	reference sequence	sequence variant	predicted consequence	LOVD	gnomAD exomes allele frequency total	gnomAD exomes allele frequency european non-finnish	ClinVar clinical significance	Classification according to genetic report (ACMG)	count
COL4A5	NM_033380.3	c.2766del	p.Gly923Valfs*73	not reported	not listed	not listed	pathogenic	p	1
COL4A5	NM_033380.3	c.2830G>A	p.Gly944Arg	1 reported	not listed	not listed	not listed	lp	2
COL4A5	NM_033380.3	c.2892_2899dup	p.Gly967Glufs*32	not reported	not listed	not listed	pathogenic	p	1
COL4A5	NM_033380.3	c.2933C>G	p.Ser978*	not reported	not listed	not listed	not listed	lp (PVS1, PM2, PP4)	1
COL4A5	NM_033380.3	c.3052G>T	p.Gly1018Cys	not reported	not listed	not listed	likely pathogenic	lp	1
COL4A5	NM_033380.3	c.3097G>T	p.Gly1033Cys	not reported	not listed	not listed	pathogenic	p	1
COL4A5	NM_033380.3	c.3152G>A	p.Gly1051Glu	1 reported	not listed	not listed	likely pathogenic	lp	1
COL4A5	NM_033380.3	c.3154C>T	p.Gln1052*	2 reported	not listed	not listed	not listed	p	1
COL4A5	NM_033380.3	c.322G>T	p.Gly108*	1 reported	not listed	not listed	not listed	lp	1
COL4A5	NM_033380.3	c.3331del	p.Thr1111Profs*41	1 reported	not listed	not listed	not listed	lp	1
COL4A5	NM_033380.3	c.3419G>T	p.Gly1140Val	not reported	not listed	not listed	not listed	p (PM1, PM2, PP3, PP4)	1
COL4A5	NM_033380.3	c.3481G>C	p.Gly1161Arg	not reported	0,0000009139	0,00000119	pathogenic	p	1
COL4A5	NM_033380.3	c.3482G>A	p.Gly1161Glu	2 reported	not listed	not listed	pathogenic	p	1
COL4A5	NM_033380.3	c.3491G>A	p.Gly1164Asp	3 reported	not listed	not listed	pathogenic	lp	1
COL4A5	NM_033380.3	c.3493G>A	p.Glu1165Lys	1 reported	0,00002008	0,00001902	VUS (3), likely benign (1)	VUS	1
COL4A5	NM_033380.3	c.3508G>A	p.Gly1170Ser	16 reported	0,00000274	0,00000119	pathogenic (6), VUS (1)	p	9
COL4A5	NM_033380.3	c.3545G>T	p.Gly1182Val	1 reported	not listed	not listed	not listed	p	1
COL4A5	NM_033380.3	c.367G>A	p.Gly123Arg	3 reported	not listed	not listed	pathogenic	lp	1
COL4A5	NM_033380.3	c.3791-41A>G	p.?	not reported	not listed	not listed	pathogenic	lp	1

Gene	reference sequence	sequence variant	predicted consequence	LOVD	gnomAD exomes allele frequency total	gnomAD exomes allele frequency european non-finnish	ClinVar clinical significance	Classification according to genetic report (ACMG)	count
COL4A5	NM_033380.3	c.384+1G>A	p.?	1 reported	not listed	not listed	likely pathogenic	p	3
COL4A5	NM_033380.3	c.3922C>T	p.Gln1308*	2 reported	not listed	not listed	pathogenic	p	1
COL4A5	NM_033380.3	c.4088-2A>G	p.?	not reported	not listed	not listed	pathogenic/ likely pathogenic	p	1
COL4A5	NM_033380.3	c.4246C>T	p.Arg1416Cys	5 reported	0,0000483	0,00005701	VUS (6), likely benign (1)	VUS	1
COL4A5	NM_033380.3	c.430G>A	p.Gly144Ser	1 reported	not listed	not listed	not listed	p	1
COL4A5	NM_033380.3	c.4315+1G>A	p.?	3 reported	0,0000009213	0	pathogenic	p	1
COL4A5	NM_033380.3	c.4414C>T	p.Arg1472Cys	4 reported	0,00001275	0,00001453	VUS	VUS	1
COL4A5	NM_033380.3	c.4425_4428del	p.Thr1476Argfs*77	not reported	not listed	not listed	not listed	p (PVS1, PM2, PP4, PP5)	1
COL4A5	NM_033380.3	c.4475G>A	p.Gly1492Asp	3 reported	not listed	not listed	not listed	lp	1
COL4A5	NM_033380.3	c.4475G>T	p.Gly1492Val	not reported	0,0000009107	0,000001188	likely pathogenic	lp	1
COL4A5	NM_033380.3	c.4495C>T	p.Gln1499*	1 reported	not listed	not listed	not listed	p	2
COL4A5	NM_033380.3	c.4575G>A	p.Met1525Ile	1 reported	not listed	not listed	not listed	lp	1
COL4A5	NM_033380.3	c.4597T>C	p.Cys1533Arg	not reported	not listed	not listed	not listed	lp (PM1, PM2, PP3, PP4)	1
COL4A5	NM_033380.3	c.466G>C	p.Gly156Arg	not reported	not listed	not listed	VUS	lp	1
COL4A5	NM_033380.3	c.4706G>C	p.Arg1569Pro	not reported	not listed	not listed	not listed	lp (PM1, PM2, PP3, PP4, PP5)	2
COL4A5	NM_033380.3	c.4786T>G	p.Trp1596Gly	1 reported	not listed	not listed	not listed	lp	1

Gene	reference sequence	sequence variant	predicted consequence	LOVD	gnomAD exomes allele frequency total	gnomAD exomes allele frequency european non-finnish	ClinVar clinical significance	Classification according to genetic report (ACMG)	count
COL4A5	NM_033380.3	c.4800G>T	p.Trp1600Cys	not reported	not listed	not listed	not listed	VUS (PM2, PP3, PP4)	1
COL4A5	NM_033380.3	c.4895C>T	p.Ser1632Leu	1 reported	not listed	not listed	VUS	lp	3
COL4A5	NM_033380.3	c.4913G>C	p.Cys1638Ser	not reported	0,000000911	0,000001188	not listed	VUS (PM2, PP3, PP4)	1
COL4A5	NM_033380.3	c.4930T>C	p.Cys1644Arg	not reported	not listed	not listed	VUS	VUS	1
COL4A5	NM_033380.3	c.5048G>A	p.Arg1683Gln	9 reported	0,000003652	0	pathogenic/ likely pathogenic	p	2
COL4A5	NM_033380.3	c.512G>A	p.Gly171Asp	not reported	not listed	not listed	not listed	lp (PM1, PM2, PP3, PP4)	1
COL4A5	NM_033380.3	c.619G>A	p.Gly207Ser	not reported	not listed	not listed	likely pathogenic	lp	1
COL4A5	NM_033380.3	c.761_762del	p.Glu254Valfs*11	5 reported	not listed	not listed	pathogenic	p	1
COL4A5	NM_033380.3	c.799G>A	p.Gly267Arg	not reported	not listed	not listed	likely pathogenic	lp	1
COL4A5	NM_033380.3	c.842del	p.Pro281Glnfs*65	not reported	not listed	not listed	not listed	lp (PVS1, PM2, PP4)	1
COL4A5	NM_033380.3	c.865G>A	p.Gly289Ser	not reported	not listed	not listed	likely pathogenic	lp	3
COL4A5	NM_033380.3	c.901G>C	p.Gly301Arg	2 reported	not listed	not listed	not listed	lp	1
COL4A5	NM_033380.3	c.904A>T	p.Lys302*	2 reported	not listed	not listed	not listed	p	1
COL4A5	NM_033380.3	DelEx38-40	N/A	not listed	not listed	not listed	not listed	p (PP4, PVS1, PM2)	1
COL4A5	NM_033380.3	DupEx42-45	N/A	not listed	not listed	not listed	not listed	lp (PP4, PVS1, PM2)	2

Gene	reference sequence	sequence variant	predicted consequence	LOVD	gnomAD exomes allele frequency total	gnomAD exomes allele frequency european non-finnish	ClinVar clinical significance	Classification according to genetic report (ACMG)	count
COL4A5	NM_033380.3	Ex3-4del	p.?	not listed	not listed	not listed	not listed	lp (PP4, PVS1, PM2)	1
COL4A5	NM_033380.3	Ex7del	p.?	not listed	not listed	not listed	not listed	lp (PP4, PVS1, PM2)	1
COL4A5	NM_033380.3	c.991-1G>A	p.?	1 reported	not listed	not listed	not listed	lp	1

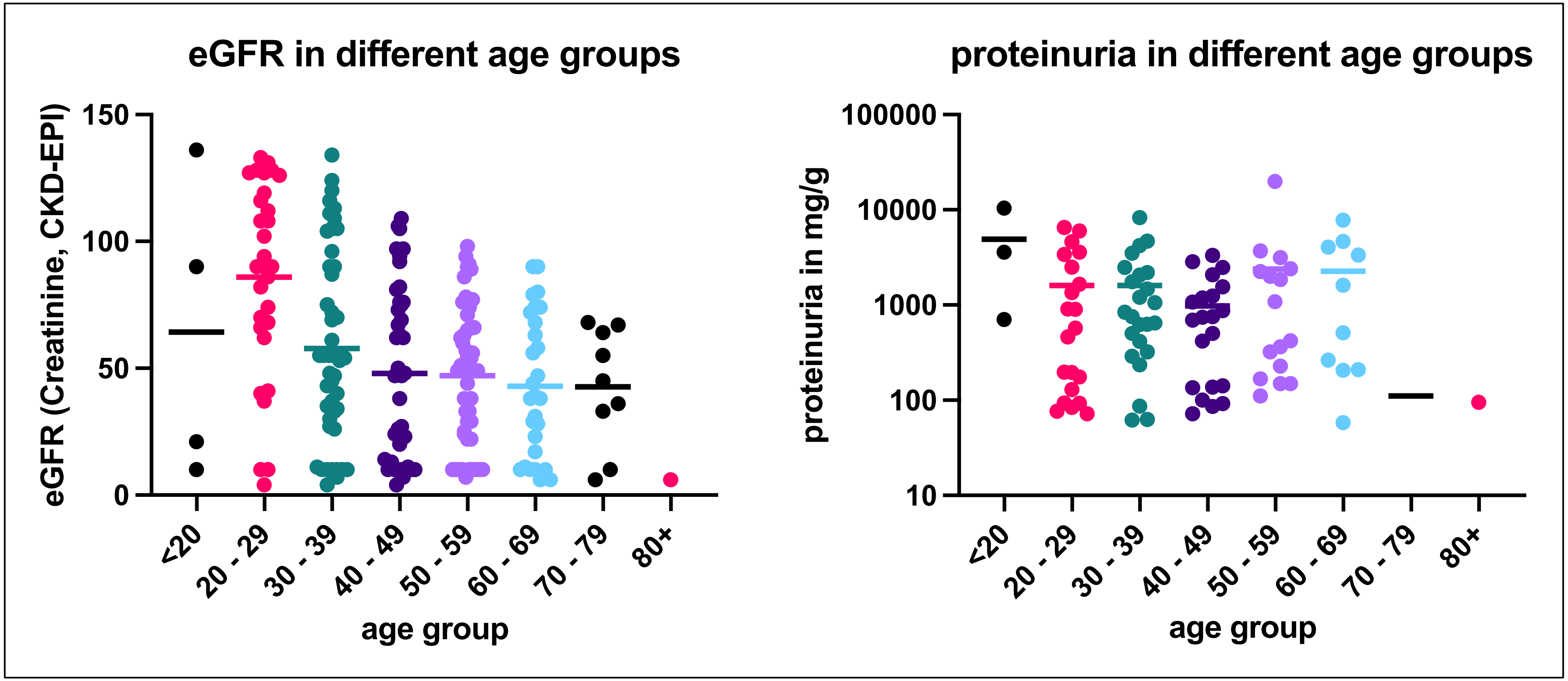

Figure S1: eGFR (left) (n = 210) and proteinuria (right) (n = 98) across age groups in the combined discovery cohort. The individual values (dots) and corresponding mean values (line) are shown.

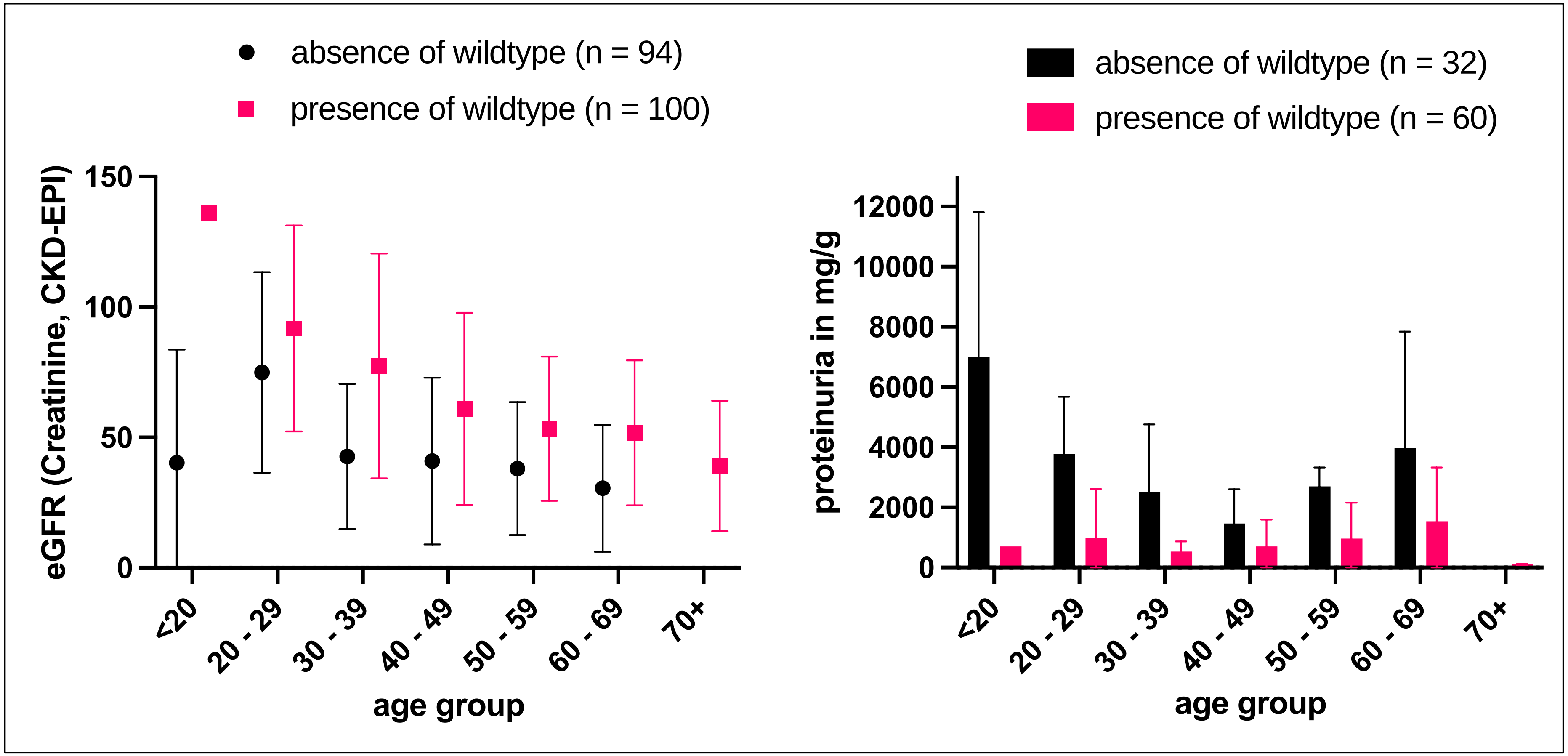

Figure S2: Comparison of mean eGFR (left) and mean proteinuria (right) with corresponding SD across age groups in patients with absence of a wildtype allele versus presence of a wildtype allele.

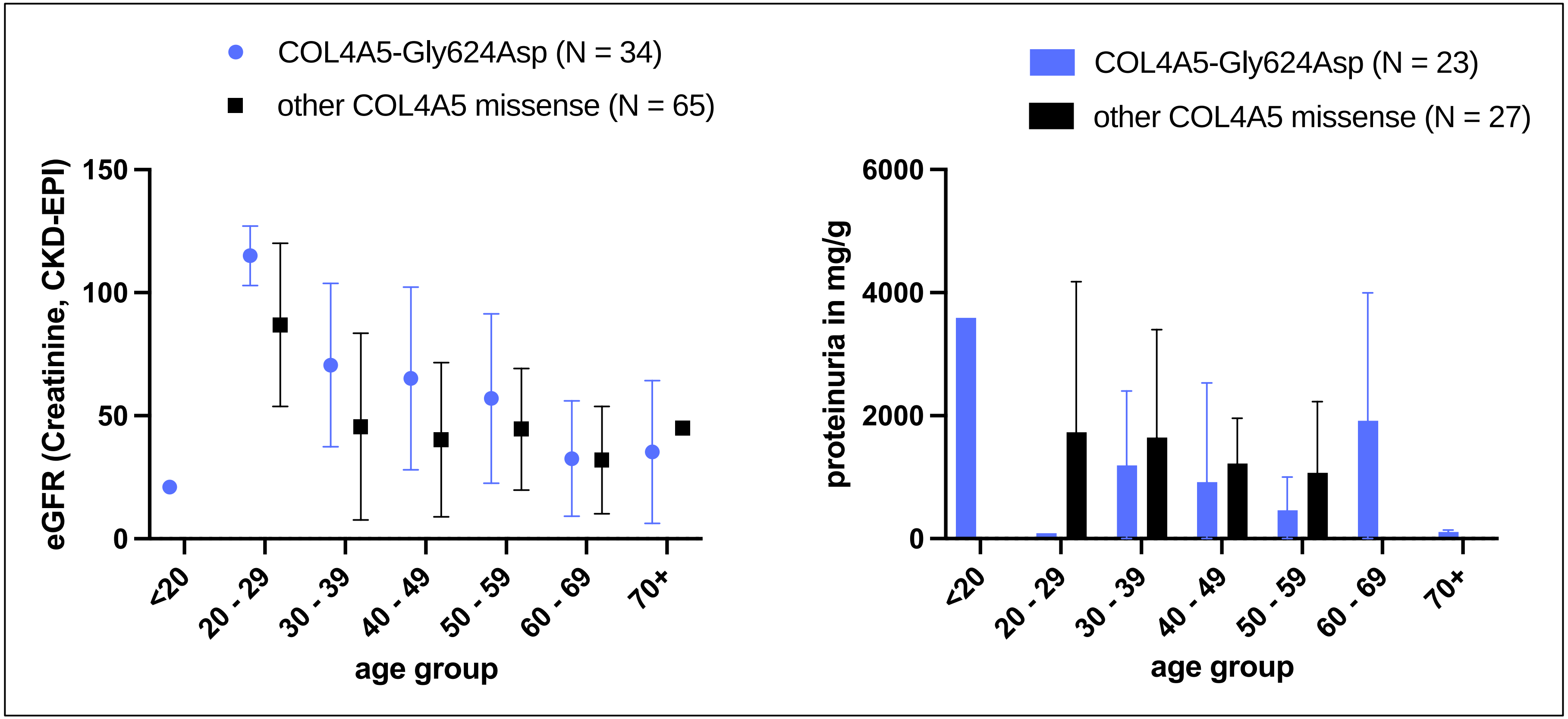

Figure S3: Comparison of mean eGFR (left) and mean proteinuria (right) with corresponding SD across age groups in patients with the *COL4A5*-p.Gly624Asp variant versus patients with other *COL4A5* missense variants .

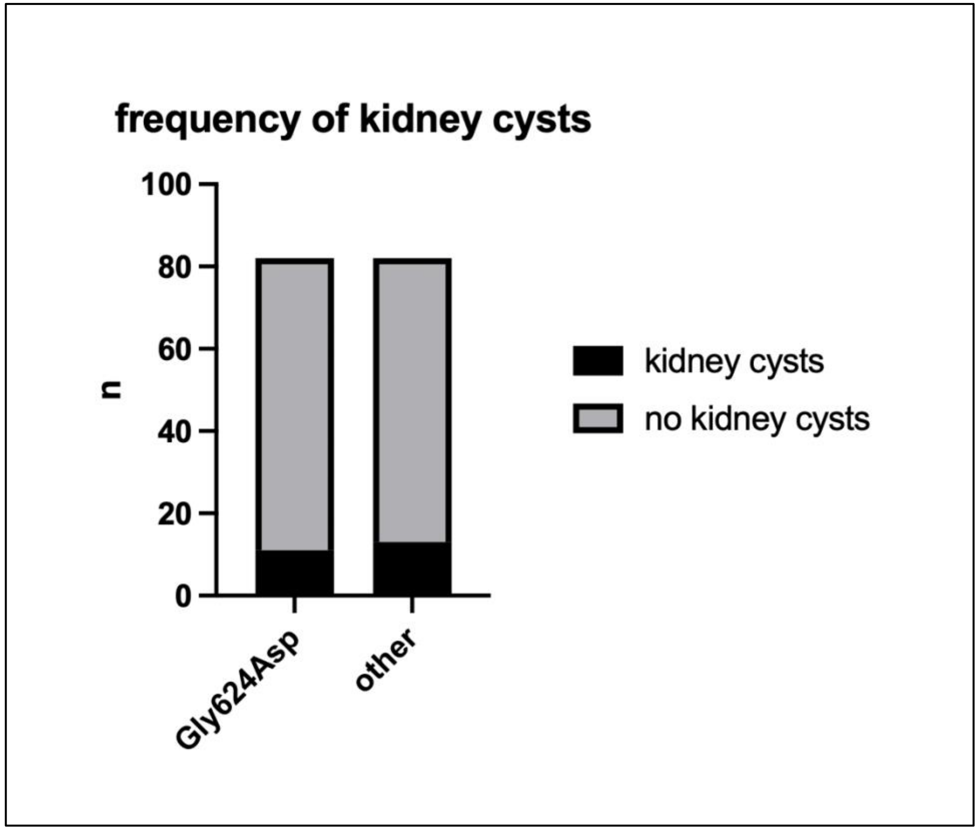

Figure S4: The overall occurrence of kidney cysts in the extended cohort did not differ between patients harboring the variant p.Gly624Asp in *COL4A5* (11/82) and patients with other Gly-missense variants in *COL4A5* (13/82).
